# The Society for Prevention Research 20 Years Later: a Summary of Training Needs

**DOI:** 10.1007/s11121-020-01151-1

**Published:** 2020-08-03

**Authors:** Sarah M. Chilenski, Keryn E. Pasch, Ashley Knapp, Elizabeth Baker, Rhonda C. Boyd, Camille Cioffi, Brittany Cooper, Abigail Fagan, Laura Hill, Leslie D. Leve, Kelly Rulison

**Affiliations:** 1 Edna Bennett Pierce Prevention Research Center, Pennsylvania State University, University Park, PA USA; 2 Department of Kinesiology and Health Education, University of Texas at Austin, Austin, TX USA; 3 Northwestern University, Evanston, IL USA; 4 Department of Psychology, University of Calgary, Calgary, Canada; 5 The Children’s Hospital of Philadelphia, Philadelphia, PA USA; 6 University of Oregon, Eugene, OR USA; 7 Washington State University, Pullman, WA USA; 8 University of Florida, Gainesville, FL USA; 9 University of North Carolina at Greensboro, Greensboro, NC USA

**Keywords:** Prevention, Training, Methodology, Workshops, Professional development

## Abstract

The Society for Prevention Research (SPR) aims to continually provide relevant professional development training opportunities to advance scientific investigation of ways to improve the health, well-being, and social and educational outcomes of individuals and communities. Our study, led by the Training Needs Assessment Task Force, designed a quantitative questionnaire informed by semistructured, qualitative interviews of 13 key prevention science informants. The questionnaire was deployed to all SPR members, of which 347 completed it. Questions about training topics were asked along 8 categories: (1) theory; (2) preventive interventions; (3) research methods, design, and evaluation; (4) teaching and mentoring; (5) practical and interpersonal skills; (6) communication; (7) project management; and (8) data analysis. Across all categories, respondents reported a high level of interest in receiving training: more than 80% were interested in training in data analytic methods; about 70% indicated interest in theory, preventive interventions, and research methods, design, and evaluation; about 65% were interested in at least 1 communication and project management topic; and 60% showed interest in at least 1 practical and interpersonal skills topic. Training-related interests varied across career level and race/ethnicity, with early-career individuals and people of color typically indicating the most interest. Participants were most likely to endorse self-initiated learning and webinars. SPR preconference training workshops were strongly endorsed for data analysis and preventive intervention topics. Recommendations from our study include a need for SPR to more strongly support self-initiated learning opportunities and continue preconference training programs, with special focuses in statistical methods and preventive interventions and regular assessment of members’ training preferences.

**Electronic supplementary material:**

The online version of this article (10.1007/s11121-020-01151-1) contains supplementary material, which is available to authorized users.

## Introduction

The mission of the Society for Prevention Research (SPR) is twofold. First, it aims to improve research and the knowledge base related to the development and prevention of social, physical, mental health, and academic/cognitive problems. Second, SPR aims to improve how this knowledge is applied to everyday situations to support health, development, and well-being (Society for Prevention Research 2018c). Given the quick pace of technological advances, supporting continual training in the foundations of SPR and new developments in the field are essential for the organization to achieve its goal, to improve the health, development, and well-being of communities across the country and around the world.

To ensure relevant training is provided to its members, the Board of Directors approved a proposal generated by Early Career Preventionist Network (ECPN) members and the training committee to survey SPR members about their training needs. As a result, a short-term task force, the Training Needs Assessment Task Force, was convened. This task force included early, mid, and senior-career SPR members. In this article, we describe the results and recommendations from the task force’s study.

### Prior Efforts to Document and Enhance the Needs of SPR Members

Two previous studies have assessed SPR member needs. The first was a web-based questionnaire, informed by qualitative interviews with 20 prevention science researchers that assessed prevention science training needs of SPR members (Eddy et al. 2005). The questionnaire, sent out in 1988, asked prevention scientists to rate their knowledge, prior training, and desire for additional training in 13 content areas. Although there were some differences according to respondent career level, the majority of participants indicated a desire for additional training in nearly every area. Véronneau and colleagues (Veronneau et al. 2012) conducted the second assessment through surveying a sample of 97 SPR members about the types of mentoring that early-career prevention scientists receive. Findings from the study highlighted the importance of mentoring, identified desirable characteristics of mentors and their protégés, and identified ways that SPR members could facilitate mentoring relationships. These findings subsequently helped guide training efforts in the following years at annual meetings.

In addition to these studies, the SPR Board of Directors approved other efforts to help prevention scientists learn about and develop expertise. One of the first initiatives was to develop standards of evidence for efficacy, effectiveness, and dissemination research (Flay et al. 2005). These standards were updated recently (Gottfredson et al. 2015). Guidelines and recommendations have also been developed on other topics such as how to develop community monitoring systems (Mrazek et al. 2006) and to conduct economic evaluations (Crowley et al. 2018). Each article outlines related standards, challenges, and training needs.

Two more SPR publications relevant to our study are the *Standards of Knowledge for the Science of Prevention* (Biglan et al. 2011) and the *Ethical Challenges in Promoting the Implementation of Preventive Interventions* (Leadbeater et al. 2018). The Biglan et al. paper outlined the foundational concepts and unique contributions of prevention science as a field of study and identified the skills and core competencies needed to be a successful prevention scientist. The Leadbeater et al. article presents principles and concepts that can be used to frame discussions about ethical concerns in prevention implementation and scale-up efforts, and summarized prevention science ethics value statements. Given their centrality to prevention science, all these areas could be targets of SPR-sponsored trainings.

### Growth and Change in the Needs of Prevention Scientists

Together, these SPR efforts provided information about the skills and knowledge important for prevention scientists to train in and master; however, the prevention science landscape has undergone significant change in the past two decades. For example, there are now multiple formal training programs in the USA that offer doctoral and master’s degrees in prevention science, and a number of programs that offer formal training, minors, or certificates in prevention science within other degree programs (Society for Prevention Research 2018d).

Similarly, prevention science as a discipline, and SPR as its leading professional organization, have continued to grow and change, increasing the number of prevention scientists, the racial/ethnic diversity of the discipline, the topic areas of study, and the types of workplaces prevention scientists call home. For example, SPR had 120 members in 1994 and 826 members in 2015 (Society for Prevention Research 2018b). SPR members now include scientists, advocates, practitioners, administrators, and policy makers at all career levels. Individuals who are new to SPR may be coming from a wide variety of fields, and thus may need training in Prevention Science foundational methods and theories. This requires well-developed skills in stakeholder communication and collaboration, especially as the demographic make-up and needs of society shifts (Feder et al. 2019).

In addition, the racial and ethnic diversity of SPR’s membership has increased 33% since 2003, from 21 to 28% of its members coming from diverse racial/ethnic populations. Preferred training modalities and topics may differ for traditionally underrepresented researchers who often face specific barriers in pursuing prevention research and funding (Franco et al. 2011; Waitzkin et al. 2006). For example, previous research has identified barriers such as greater university service responsibilities, having less access to research facilities, and having limited mentorship availability by researchers of color (Alegria et al. 2019; Hemming et al. 2019; Hoppe et al. 2019; Settles et al. 2020). These barriers, among others, can negatively impact the research career trajectory and perpetuate limited representation of people of color in prevention research (Lee et al. 2012).

The past decade has also seen an explosion in new prevention methodologies and approaches (e.g., ecological momentary assessments, adapted intervention designs, genomic, and neuroimaging data collection methods; Brown et al. 2017; Brown et al. 2014; Collins 2018; Schuster et al. 2016; Wiedermann et al. 2019) and, correspondingly, a need to train individuals at all career levels in these new methods. Technological resources and capabilities have also grown exponentially, enabling innovative data collection and analysis techniques (Mason et al. 2015; Mohr et al. 2017; Ridenour 2018; Schuster et al. 2016; Torous et al. 2018). Clearly, the state of science is rapidly evolving, underscoring the need to re-assess the membership of SPR to gauge training needs with regard to new methods and innovative approaches to keep the field current.

### The Current Study

These trends prompted the Training Needs Assessment Task Force to update the current training needs of SPR members in foundational skills, newly emerging topics, collaboration, translational research, teaching, and mentoring. Thus, the first aim was to descriptively report trends in reported training needs for the full sample. Then, our second aim was to examine whether training needs differ by career level, race/ethnicity, and their interaction. We expect that individuals early in their career will have less knowledge, and thus have more training needs than those who are further established (Eddy et al. 2005). However, given the growth and change in prevention science, it is possible that established researchers lack training in emerging areas. Thirdly, given the historic underrepresentation and barriers of researchers of color in the field, we expect that the training needs of researchers of color will likely be greater.

## Methods

### Participants

To maximize the respondent pool, the task force emailed questionnaire invitations to anyone who was an SPR member in 2016 and to SPR members in 2017 as of May 25, 2017 (*n* = 1229 individuals), as well as to the SPR listserv. Although 396 individuals started the questionnaire, 49 did not continue past the initial consent screen, resulting in a final sample of 347 respondents (28.2% response rate). This response rate is higher compared with other online questionnaires distributed to members of national professional organizations (e.g., 13.0–17.5%; Veronneau et al. 2012; Yetter and Capaccioli 2010) and similar to prior SPR surveys. Further, it is comparable to a recent survey of all members of the American Society of Breast Surgeons (Zhang et al. 2019). All but 20 respondents indicated that they were a member of SPR at the time they took the questionnaire (94.2%). The majority of respondents identified as female (73.4%) and White (72.2%). A range of other race/ethnicities was also reported (1% American Indian/Native American; 8.3% Asian/Pacific Islander; 7/6% Black/African American; 5.0% Spanish/Hispanic/Latino; 6.0% multiracial). Most respondents (68%) had earned a doctoral degree and almost two-thirds of respondents were early in their careers (60.3%). Overall, while 20% of the respondents were both of color and early career, 72% of the respondents of color were early career, while 56% of early-career respondents were people of color. The Penn State Institutional Review Board approved the study as exempt before participant recruitment began.

### Procedures

#### Questionnaire Design

The task force identified eight sections for the questionnaire based on the main themes that emerged from key informant interviews (for the protocol and results see supplemental material S1, available online). Each task force member developed content for one section, drawing on SPR’s Strategic Goals (Society for Prevention Research 2016), SPR’s Standards of Knowledge (Biglan et al. 2011), SPR’s membership intake form (Society for Prevention Research 2018a), and previous training-focused publications (Eddy et al. 2005; Tabak et al. 2017; Veronneau et al. 2012). The language and scope of the items were refined collaboratively by the entire task force. Similar to Eddy et al. (2005), we asked the same series of questions about participation likelihood and preferred training modality for all sections. The SPR Board of Directors approved the final questionnaire.

#### Data Collection

The task force programmed the questionnaire in Qualtrics and emailed a unique link to 2016 and 2017 SPR members just before the 2017 conference. We emailed three reminders during the following 6 weeks, with one additional reminder emailed in August. The unique links allowed us to send targeted reminders to anyone who had not yet completed the questionnaire. In response to concerns about spam blocks for Qualtrics emails, we also sent the anonymous questionnaire link to the SPR listserv shortly after sending each reminder. The questionnaire took 15–20 min. After completion, respondents had the chance to enter a raffle for one of three free SPR memberships or one of 40, $20 Amazon gift certificates.

### Measures

#### Questionnaire Items

The questionnaire itself (see supplemental material, S2, available online) had eight main sections: (1) theory; (2) preventive interventions; (3) research methods, design, and evaluation; (4) teaching and mentoring; (5) practical and interpersonal skills; (6) communication; (7) project management; and (8) data analysis. Within a section, respondents indicated how likely they were to participate in training for each topic by selecting from “1” (*not at all—I already have adequate knowledge or skills in this area*), *“*2” (*not at all—this area is not relevant to me*), “3” (*somewhat likely*), or “4” (*very likely*). If a respondent selected either *somewhat likely* or *very likely* for one or more of the associated topics within a section, they then indicated their preferred training modality for that section: (a) self-initiated learning, (b) one-on-one mentoring/coaching/consulting, (c) webinar, (d) SPR preconference workshop, (e) 1-day in-person workshop (not SPR conference affiliated), and (f) experiential education. Respondents could select multiple preferred training modalities. The teaching, mentoring, quantitative, and qualitative data analysis sections included an option to skip these sections if they were not relevant for the respondent. An introductory statement instructed respondents to think beyond the current training available to them and to consider all their training needs. This statement also reminded respondents that training could take many forms, from self-initiated learning (e.g., reading materials, watching presentations on your own), to one-on-one mentoring opportunities, webinars, preconference or other workshops, or experiential learning opportunities.

#### Indices

Two indices combined topics across categories to estimate training needs as a whole. The first index added all training topics together to create an *overall training interest score* of the likelihood of participating in training. Topics that were endorsed as “very likely” were given the value of “2,” endorsements of “somewhat likely” were given a “1,” and “not at all” responses were given a “0.” This scale is sensitive to how strong the likelihood is that a respondent will participate in training (*M* = 68.31, SD = 40.11). The second index, a simple *count of training topics*, simply counted the number of items endorsed with “somewhat likely” or “very likely” as a “1” and all other responses as “0” (*M* = 52.18, SD = 27.27).

#### Demographics

At the end of the questionnaire, respondents completed most of the demographic questions asked on the SPR membership intake form including self-identifying gender, race/ethnicity, highest degree, and career level (see full questionnaire in Supplement 2, available online). From this information, we created a respondent type variable: early career (undergraduates, graduate students, postdoctoral fellows, first-year professionals, and early-career professionals), midcareer, and senior career. For the descriptive analyses, we examined mid- and senior-career respondents separately. However, for the regression analyses (see Data Analysis subsection), we collapsed the mid- and senior-career respondents to create an *early-career status* variable (0 = not early career included mid and senior; 1 = early career, included all others). Given, the large percent of participants endorsing *White*, *Caucasian*, *European* (71.1%), and the uneven and small distribution of participants in other race/ethnicity categories (range 1.0 to 9.2%), we recoded the race/ethnicity items into White (0) and people of color (1).

### Data Analysis

#### Preliminary Analyses

We obtained simple frequencies to review sample demographic characteristics and compared them with the full 2017 SPR membership sample. We did not conduct significance tests because the purpose was to describe general patterns.

#### Analysis of Training Topics

We obtained simple frequencies for the “somewhat likely” and “very likely” responses for each topic within a questionnaire section. We then obtained frequencies as a function of career level (i.e., early career, midcareer, and senior career) and race/ethnicity (i.e., people of color vs. White). As with preliminary analyses, we did not conduct significance tests because the purpose was to describe general patterns.

#### Regression Analyses

After noting similar patterns between early-career status and people of color, we conducted regression analyses to determine whether the effects of early-career status were independent from race/ethnicity. The dependent variables in the regressions were the indices described in the measures section. Dummy codes for early-career status and race/ethnicity, along with the interaction between the two, were entered as predictors.

## Results

### Representativeness of the Sample

The final study sample reflected the full 2017 SPR membership with a few exceptions. The study sample included a higher percentage of early-career respondents (see Table 1) and a slightly lower percentage of Latino respondents and respondents with a doctoral degree. Further, multiracial and female respondents had slightly higher levels of participation.

**Table 1:** Respondent Demographics Compared With the Demographics of the Full 2017 Membership of SPR

	% survey sample* N = 347	% 2017 membership roster *N* = 742
Gender		
Male	26.6	31.9
Female	73.4	68.1
Race/ethnicity		
American Indian, Native American	1.0	1.7
Asian, Pacific Islander	8.3	8.8
Black, African American	7.6	7.7
Spanish, Hispanic, Latino	5.0	9.2
White, Caucasian, European	72.2	71.7
Multiracial	6.0	1.0
Race/ethnicity dichotomized		
White	72.2	71.7
People of Color	27.8	28.3
Highest degree		
High school	0.1	0.0
Bachelor’s	7.1	7.1
Master’s	24.4	20.3
PhD/MD/JD/EdD/other doctoral degrees	68.2	72.6
Career level		
First-year professional	3.5	2.3
Student	19.3	21.9
Early career	29.3	22.5
Postdoc	7.0	5.8
Midcareer	21.9	23.8
Senior career	17.0	23.7
Other	1.9	2.4
Career level dichotomized		
Early career	60.3	52.8
Mid- or senior career	39.7	47.2

*Due to item-level missing data, the sample size ranged from 302 to 311 across items, with exception of the membership question, which had *n* = 208 because that item was added to the survey after the survey was launched

### Analysis of Training Topics

Overall, respondents reported a high level of interest (defined as “somewhat likely” or “very likely” to participate) in continued training. On average, respondents indicated interest in 52 (out of 116) training topics (min = 0; max = 115; SD = 27.27). Notably, there was sizeable interest in at least one topic in each section, although there was some variability in interest across sections. Just more than 80% of respondents indicated interest in at least one data analytic methods topic. About 70% of respondents indicated that they were interested in one or more topics in the theory; preventive interventions; and research methods, design, and evaluation sections. By contrast, 65% of respondents were interested in at least one communication-related and project management–related topic, and 60% of respondents were interested in at least one practical and interpersonal skills topic. Training interest related to teaching and mentoring was also of high interest among those who consider teaching or mentoring to currently or will in the future be part of their regular job responsibilities. Interest in training often varied across career level and race/ethnicity. We describe specific results for each section below.

### Section 1: Prevention Science Theory

More than half of respondents indicated interest (i.e., “somewhat likely” or “very likely” to participate) in each theory-related topic (see Table 2). The most popular topics included mechanisms for addressing health disparities (79%), the role of context in shaping health behavior (73%), complex systems and systems theory (71%), and theories of change (70%). See supplemental information S3 (available online) for subgroup analyses. Specific subgroup analyses revealed that early-career respondents indicated more interest than did mid- and senior-career respondents in all theory-related topics, except for etiology/epidemiology of health behaviors. People of color indicated more interest than did White individuals for all theory-related topics except for complex systems. Notably, however, mechanisms for addressing health disparities was the most popular topic across all subgroups.

**Table 2:** Overall results for all survey subsections

	% somewhat or very likely participate
Prevention science theory topics (*n* = 298–301)	
Mechanisms for addressing disparities	79
Context shaping health behavior	73
Complex systems and systems theory	71
Theories of change	70
Key principles in public health	68
Etiology/epidemiology of health behaviors	63
Foundations of prevention science	59
Human developmental theory	55
Preventive intervention topics (*n* = 329–332)	
Incorporating new technologies	85
Consideration of cultural competency	81
Understanding the negative effects of interventions	81
Dissemination research	79
Implementation research	78
Targeting prevention interventions to reduce health disparities	76
Community input and collaboration	71
Recruiting, engaging, and retaining participants	69
Creating materials and guidelines for intervention delivery	65
Developing intervention logic models	64
Training and technical assistance	61
Effective and ineffective interventions	59
Research methods, design, evaluation topics (*n* = 320–322)	
Hybrid designs combining effectiveness & implementation	79
Adaptive intervention design	77
Mixed or multimethod hybrid qualitative/quantitative	73
Longitudinal design	73
Nonexperimental design/quasi-experimental design	71
Survey sampling methods	63
Experimental design	61
Data management	56
Data collection & survey; nonnative English speakers/nonliterate populations	55
Biological & physical data collection & analysis	46
Ethical practices	41
Mentoring topics (*n* = 261–263)	
Giving constructive criticism and feedback	66
Applying for external grant funding	64
Successfully guiding students through the undergraduate or graduate thesis or PhD dissertation process	59
Job-search and interview skills for both academic and nonacademic positions	59
Collaborating as part of a team	58
Establishing a mentoring relationship	53
Teaching topics (*n* = 210–216)	
Active learning strategies	77
Skills for discussing hot-button issues	73
Increasing student engagement	69
Assessment methods	67
Strategies for teaching online courses	67
Effective use of teaching assistants	61
Developing course materials	56
Practical and interpersonal skills (*n =* 302–308)	
Networking in prevention science	65
Initiating community collaborations	65
Initiating interdisciplinary collaborations	64
Stress management, work–life balance	57
Time management	56
Working with others with different backgrounds; maintaining diverse teams	54
Maintaining motivation with obstacles	52
Developing and maintaining collaborations	50
Receiving constructive feedback	45
Communication-related skill topics (*n* = 293–304)	
Communicating your work to the general public via various social media platforms	74
Communicating with government officials	70
Communicating research to lay audiences	70
Communicating with foundations	67
Communicating your work to peers, colleagues, or funders via various social media platforms	64
Writing manuscripts for peer reviewed journals	47
Presenting at professional conferences	39
Project management topics (*n =* 299–302)	
Understanding potential funding opportunities	71
Evaluating project outcomes	70
Effective leadership	64
Developing and managing budgets	64
Understanding contract/grant requirements	63
Developing and managing schedules, timelines, expectations, deliverables, and quality	62
Recruiting, hiring, managing, and mentoring project staff	57
Meeting management	52

### Section 2: Preventive Interventions

Respondents indicated the most interest in training about preventive intervention topics that represent newer areas of research (see Table 2). More specifically, the most popular preventive intervention topics were incorporating new technologies into the design, implementation, or evaluation of interventions (85%); consideration of cultural competency in designing, delivering, and adapting interventions (81%); and understanding the unintended negative effects of interventions (81%). Specific subgroup analyses (S3, available online) revealed that early-career individuals and people of color indicated more interest in each preventive intervention topic. Incorporating new technologies was the most highly rated topic in all subgroups, with dissemination research, implementation research, and targeting preventive interventions to reduce health disparities also highly endorsed in each subgroup. Many early-career respondents were also interested in soliciting community input and collaboration.

### Section 3: Research Methods, Design, and Evaluation

Respondents indicated the most interest in integrative and interdisciplinary research method topics (see Table 2). Most popular topics were hybrid designs combining effectiveness and implementation (79%), adaptive intervention design (77%), mixed or multimethod hybrid designs (73%), longitudinal design (73%), and nonexperimental and quasi-experimental design (71%). Fewer than half of respondents expressed interest in biological and physical data collection and analysis (46%) and ethical practices (41%). Specific subgroup analyses (S3, available online) revealed that early-career individuals and people of color indicated more interest than did their counterparts in all research methods topics, except for ethical practices, which was endorsed at similarly low rates across career levels. The top five research method training interests were consistent across career levels and racial/ethnic groups.

### Section 4a: Mentoring

In this section, respondents answered a screener item about their mentoring experience. Respondents who selected *currently mentor now* or *expect to mentor in the future* then answered questions about their needs for training with respect to mentoring (*n* = 262). There was little variability in interest across mentoring topics: Between 53 and 66% of respondents expressed interest in each topic (see Table 2). The most popular topics were giving constructive criticism and feedback (66%) and applying for external grant funding (64%). Specific subgroup analyses (see S3, available online) revealed that early-career individuals and people of color indicated the most interest in all mentoring topics. Learning how to mentor others who are applying for external grant funding was the most popular topic for both early-career and senior-career respondents, as well as for both people of color and White respondents. By contrast, giving constructive criticism and successfully guiding students through the thesis/PhD process were the most popular topics for midcareer respondents.

### Section 4b: Teaching

In this section, respondents answered a screener item about their teaching experiences. Those who selected *currently teach now* or *expect to teach in the future* then answered questions about their needs for training with respect to teaching (*n* = 216). Each teaching topic was endorsed by more than half of the respondents who either had teaching experience or expected to teach in the future (see Table 2). Respondents indicated the most interest in how to enhance student interest and engagement. Specific subgroup analyses (see S3, available online) revealed that early-career individuals and people of color reported the highest levels of interest in all teaching topics. Discussing hot-button issues was the most popular topic for early-career and White respondents, and it was tied with active learning strategies as the most popular topic for people of color. Active learning strategies was also the most popular topic for midcareer respondents and tied with strategies for teaching online courses for senior-career respondents.

### Section 5: Practical and Interpersonal Skills

Respondents indicated the most interest in practical and interpersonal trainings about building relationships with possible collaborators (see Table 2). Specifically, the most popular topics were networking or building/maintaining connections in the prevention science community (65%), initiating collaboration with the general community (65%), and initiating interdisciplinary collaborations (64%). Specific subgroup analyses (see S3, available online) revealed that early-career individuals and people of color indicated the most interest in practical and interpersonal skills training. Initiating community collaborations was the most popular topic among early-career respondents, White respondents, and people of color. By contrast, stress management/work–life balance was the most popular topic for midcareer respondents.

### Section 6: Communication

Respondents indicated the most interest in training focused on communicating to non-prevention research audiences (see Table 2). The most popular topics were communicating work to the general public via various social media platforms (74%), communicating research to lay audiences (70%), and communicating with government officials (70%). Specific subgroup analyses (see S3, available online) revealed that early-career individuals and people of color indicated the most interest in communication trainings. There was variation across career levels, however, in terms of which audience respondents wanted to focus on: Early-career respondents were most interested in learning about communications with government officials, midcareer respondents were most interested in communicating to the general public via social media platforms, and senior-career respondents were equally interested in communicating to lay audiences and with peers, colleagues, or funders via social media platforms.

### Section 7: Project Management

All project management topics were endorsed by more than half of the respondents (see Table 2). The most popular topics were understanding potential funding opportunities (71%) and evaluation of project outcomes, such as use of “dashboards” and other tools that serve as a metric for progress and success (70%). Specific subgroup analyses (see S3, available online) revealed that early-career respondents indicated the most interest for all eight project management topics. Similarly, people of color indicated more interest in all project management topics. Understanding potential funding opportunities was the most popular topic among early and senior-career respondents and White respondents. Midcareer respondents were most interested in evaluating project outcomes, and people of color preferred training about budgets (44%).

### Section 8: Data Analysis

In this section, respondents answered two screener items about how likely they were to participate in training related to (1) quantitative methods and (2) qualitative methods. Those who indicated *somewhat likely* or *very likely* for quantitative methods received a list of 41 statistical methods (e.g., Bayesian methods, growth modeling). Those who indicated *somewhat likely* or *very likely* for qualitative methods received a list of seven qualitative methods. Respondents then indicated if they “would likely attend training” in each method. They could also indicate “statistical method unknown” if they were unfamiliar with that method.

Most respondents (81%) indicated that they were interested in training for quantitative analytic methods. The top 10 quantitative topics are listed in Table 3, with a full list available in supplemental material S4 (available online). The most popular was cost-effectiveness methods (63%), followed by other techniques used for analyzing longitudinal data and other complex data structures. Only 18% of respondents indicated interest in alternative/authentic assessment, but this might be explained by lack of familiarity with this method: 48% of respondents indicated that this method was unknown to them. Specific subgroup analyses (see S3, available online) revealed that early-career respondents indicated the most interest in quantitative methods and midcareer respondents in qualitative methods. People of color indicated more interest in both quantitative and qualitative methods than did White respondents.

**Table 3:** Overall analyses for quantitative (top 10 endorsed) and qualitative data analysis section

Quantitative analysis topics (*n* = 263*)*	% likely attend	% method unknown
Any quantitative analysis (*n* = 325)	81	–
Cost-effectiveness methods	63	11
Statistical power analysis	54	5
Intensive longitudinal data analysis	54	8
Causal inference	54	13
Propensity score methods	54	16
Mixture models	53	8
Analysis of small sample data	52	8
Growth modeling	49	10
Meta-analysis	49	7
Missing data analysis	49	7
Qualitative analysis topics (*n =* 214)	% likely attend	% method unknown
Any qualitative analysis (*n* = 325)	66	–
Focus groups	66	3
Content analysis	65	6
Key informant interviews	58	
Structured observation	54	5%
Case studies	44	6
Document analysis	39	13
Alternative/authentic assessment	1	48

### Preferred Training Modalities

Across all sections except data analyses, the most preferred modalities for training were self-initiated learning (range 66–72%) and webinars (64–78%). Many respondents also indicated an interest in SPR preconference training for preventive intervention topics (61%). The most preferred modalities for training in data analyses were evenly divided across SPR preconference workshop (62%), webinar (60%), and self-initiated learning (59%). See supplemental material S5 (available online) for more information.

### Qualitative Item

To help the task force and the SPR Board interpret the findings, respondents answered one open-ended item at the end of the questionnaire to capture any remaining ideas related to training and SPR. This question was, “What other insight or perspective can you share to help us understand your training needs and the likelihood that you will take advantage of future training opportunities organized by the Society for Prevention Research?”

Of the 347 questionnaire respondents, 86 answered this item (24.8%). A team of three prevention science students (two graduate, one undergraduate) used emergent coding to identify nine main themes: (a) across academic career levels, affordability is important to attend trainings; (b) additional accommodation for members with limited institutional or personal resources, or from rural areas is needed to support participation; (c) web-based trainings would be useful across all career levels; webinars that can be archived and viewed later would be beneficial for members that are unable to make conferences; (d) SPR should accommodate international members or those outside the USA; (e) research method training is important across career levels; respondents requested specific topics in both quantitative and qualitative methodologies; (f) graduate students and postdoctoral fellows expressed interest in job, network, and collaboration opportunities for professional development and transitional periods (e.g., Ph.D. to professorship); (g) early-career professionals are more likely to request training in a variety of areas; (h) midcareer respondents emphasize time is a barrier; and (i) senior-career respondents are less likely to request training and report perceiving a stronger emphasis on training earlier career students and professionals and bridging the gap between research and practice. Full results with examples are located in the supplemental material S6 (available online).

### Regression Analyses

We conducted regression analyses to determine whether early-career status and race/ethnicity independently predicted likelihood to participate in training. Consequently, we entered early-career status and race/ethnicity in a regression model predicting the *overall training interest score* and the *count of training topics* scales. We also tested an interaction between the two independent variables.

To better understand the intersection between race/ethnicity and career status, a model was run to determine if there was a significant interaction between these two variables. The interaction was not significant so the main effects of early career and race/ethnicity were examined and each were found to contribute uniquely to training interest. Early-career respondents were significantly more interested in training than were non-early-career respondents (i.e., mid- and senior-career respondents; range from *B* = .39 to *B* = .40; all *p*s < .001) for both dependent variables. In addition, people of color were significantly more interested in training than were White individuals (range from *B* = .13 to *B* = .25; all *p*s < .05).

## Discussion

The questionnaire sent to SPR members produced three main findings: First, members indicated a strong interest in training across all eight areas. Second, early-career individuals and people of color consistently reported the most interest in training. Finally, respondents said they preferred webinars and self-initiated learning opportunities for most topics, although they were also highly interested in preconference workshops for preventive interventions and data analytic methods. These findings reflect the changing nature of prevention science, the growth and change in the membership of SPR, the emergence of prevention science–specific training programs, and the continual advancements in technology and in research and statistical methods.

### Strong Interest in Training

Respondents expressed a strong interest in training across all topics; although they were particularly interested in several areas that reflect the continued growth of prevention science as a discipline (Gottfredson et al. 2015), underscoring the necessity of ongoing training for prevention scientists. These new areas included emerging topics in research and teaching (e.g., incorporating new technologies across research stages) (Spoth et al. 2013; Tabak et al. 2017), topics related to health equity and cultural competence (e.g., mechanisms for addressing disparities) (Alegría 2009; Norris and Agodoa 2005), different intervention designs (e.g., hybrid designs combining effectiveness and implementation) (Brown et al. 2017), and communicating and collaborating with different audiences (e.g., communicating with the general public via social media platforms). SPR has offered training in some of these areas through preconference workshops or other publications (Crowley et al. 2018; Saltz et al. 2005; Society for Prevention Research 2016). Given results of this study, training in these areas should be continued and expanded to the other areas mentioned.

Of note, almost three-quarters of respondents were early career which may have contributed to the higher levels of interest in training. Although early-career prevention scientists may have more access to learning opportunities to use new and emerging methods through doctoral and postdoctoral training programs than would mid- or senior-career prevention scientists (Society for Prevention Research 2018d), similar to prior work (Eddy et al. 2005), the results suggest that early-career professionals are more likely to endorse a strong interest in training across almost all areas. Exceptions include training in epidemiology, etiology, and health behaviors, which were lower across all career levels. This lack of interest in further training in these areas may represent the strong knowledge and skills base many prevention scientists have in these foundational areas of our discipline (Gottfredson et al. 2015). This is especially encouraging given the increase in prevention science programs over the years (Society for Prevention Research 2018d).

After accounting for early-career status, respondents of color were still significantly more likely to endorse interest in training. It could be that with the increase in diversity of SPR members, those who are new to the discipline are interested in these training topics. This endorsement of training may be an accurate reflection of the support researchers of color feel they need given the barriers they face in pursuing prevention research and funding (Franco et al. 2011; Waitzkin et al. 2006). Additional training support may be especially needed given the limited representation of people of color in prevention research (Lee et al. 2012), and other barriers found in prior research such as greater university service responsibilities, less access to appropriate research facilities, and few mentors of color (Alegria et al. 2019; Hemming et al. 2019; Hoppe et al. 2019; Settles et al. 2020).

One area in which midcareer respondents expressed the most interest in training was in qualitative data analysis. Perhaps the importance of qualitative data becomes more apparent in midcareer, or midcareer researchers find that they have more time to invest in qualitative research after tenure has been granted or they have become more established. Alternatively, it is possible that qualitative methods were not well represented in past training programs, and midcareer researchers desire training in these methods as the field shifts to more greatly value mixed methods. To our knowledge, few recent SPR preconference workshops have focused on qualitative methods; as such, the training committee may want to invite proposals for qualitative method topics for upcoming conferences. Alternatively, webinars may be another opportunity for training; respondents were also interested in this modality for learning data analytic methods.

The growth in prevention science has resulted in multiple formalized training programs. The increase in prevention science–specific degree programs has renewed focus on teaching and mentoring, longstanding areas of importance to the discipline (Veronneau et al. 2012). All subgroups indicated an interest in training in these areas, but interest was most pronounced among early-career respondents and people of color, particularly in the areas of hot-button issues (for early-career respondents) and active learning strategies (for people of color). Notably, interest in training to support teaching was more heavily endorsed. However, because few training opportunities in mentoring exist, we recommend increasing opportunities in this area.

Ethics in prevention science are gaining increasing attention, and the SPR MAPS task force on ethical challenges in prevention science described this focus in a recent publication (Leadbeater et al. 2018). Research and prevention interventions are becoming more complex given increasing globalization, technology use, and the ease with which “big data” can be captured, analyzed, and shared without an individual fully recognizing the possible risks or benefits of sharing information. Further, individuals may not be aware that their information is being shared and possibly used in behavior-guiding or nonbeneficial ways (Yurieff 2018). Although clearly, training in ethics is needed, our study revealed only moderate interest in this area. We did not assess how much ethics training individuals receive. It is possible that individuals are confident that they already possess the skills and resources to conduct research and act in an ethical manner. It could also be that individuals think of ethics training more narrowly as the standard training required in our field (e.g., the Collaborative Institutional Training Initiative, CITI) (CITI Program n.d.), rather than the spectrum of ethical issues that can arise when translating research to practice. We need to consider many types of ethical issues when we place prevention science projects in community settings.

New assessment and intervention methodologies used in prevention science require the use of unique privacy and confidentially principles that are not covered in basic ethics training courses; individuals using these technologies need to be trained in relevant areas to ensure that the rights of human research participants are upheld. Identifying where to begin or how to access credible training resources may be problematic because of the novel and vast nature of concerns. As a result, despite the somewhat tepid endorsement by our respondents, we suggest that SPR lead in supporting high-quality ethical decision making by prevention scientists. Compiling an edited book or collection of articles that can be used as curriculum in undergraduate and advanced educational programs could be helpful. Adding an “ethics-” themed keynote presentation related to the unique ethical considerations of the conference theme at each SPR conference may reach a broad audience. A resource assessment of existing ethics training programs, texts, or courses may also be valuable. Certainly, these ideas are just a few.

### Preferred Training Modalities and Design

Webinars and self-initiated learning opportunities (e.g., reading materials, watching presentations) were the most frequently endorsed training modalities. A preference for self-initiated learning methods poses some difficulties: their quality varies, and it is difficult to track their use and impact. Information on quality, use, and impact are necessary for potential users and training funders to make informed decisions. How can we ensure quality? Perhaps continuing education credits or certifications can be received once mastery of a topic has occurred. While a paucity of research exists on preferred training modalities for professional organizations, one group found physiotherapists expressed the most interest in workshops, seminars, mentoring, and coaching for sports psychology training (Arvinen-Barrow et al. 2008). This suggests preferred training modality may vary by organization.

Changes in technology more easily support individual learning and broaden access to training resources. Institutions have web access to journals, and many journals have open access. Many webinars are provided free of charge, and online software is also free. These training modalities can facilitate faster development of training opportunities that may be particularly suited for emerging topics with fast dissemination needs. These modalities may also offer more equitable and inclusive access to people of color, early-career, and international scientists.

The interest in self-initiated learning is timely given SAMHSA’s investment in regional prevention training and technical assistance centers (Substance Abuse and Mental Health Services Administration 2018). These regional centers are required to evaluate trainings, addressing concerns about tracking and quality. SPR could replicate and expand Penn State’s One-and-One series. In this program, experts facilitate a workshop/webinar, and each attendee receives a 1-h follow-up discussion with the expert. This training model can also be tracked.

Although online and self-directed modalities are useful and were strongly endorsed in our study, respondents nevertheless expressed a desire for workshops and in-person trainings. These training modalities may facilitate more focused, in-depth study than independent learning. Respondents were interested in preconference workshops for training in analysis and preventive intervention methods. The SPR training committee could consider organizing workshops in this area going forward. Although respondents did not frequently report a need for mentoring opportunities and experiential education, these areas may be of great value. During SPR’s early years, programs such as those that connected senior investigators with junior investigators resulted in what some participants in our roundtable event called “pivotal experiences,” and helped establish long-lasting collaborations and successful career trajectories. Because of this, we recommend bringing back some experiential learning opportunities to the annual conference.

Changes related to the delivery of trainings may also be useful to improve access to early-career professionals and researchers of color. For example, recruiting trainers that come from a variety of racial/ethnic backgrounds will further SPR’s commitment to diversity as well as engage researchers of color. Locations of in-person trainings can also be prohibitive if travel is cumbersome and costs are high; improving access in these ways is likely to improve access to all members and particularly early-career members who may be limited in their ability to travel.

Finally, recent reports (e.g., Togami et al. 2018) have emphasized the importance of training in common competencies that cuts across disciplines, such as grantsmanship, collaboration, ethics, and communication, topics that were also frequently mentioned. One potential future direction for training opportunities could be to consolidate training on these topics for early-career scientists across disciplines or professional organizations. Other research has shown that individuals in the sciences are well trained in their content area but lack expertise in the skills and competencies that cut across disciplines (Tabak et al. 2017; Togami et al. 2018). Organizations may do well to share training opportunities in these common competencies.

### Summary

Overall strong interest was shown in training, particularly from early-career respondents and people of color. Foundational areas, such as research methods and data analysis, remain greatly favored. Emerging areas, such as communicating on social media, initiating interdisciplinary collaborations, and cost-effectiveness analysis, also garnered high interest.

### Implications

Data from our study produced a wealth of information that SPR and related prevention science training programs can draw on to further design and improve training programs. In fact, one former result from the previous training needs assessment was the shared posting of prevention science-related syllabi. Similarly, several next steps are possible and some next steps have already began, such as informing the selection for the annual preconference workshops.

We described the most salient ideas. We encourage SPR and prevention science training programs to further review and discuss these findings. To successfully meet the training needs of prevention scientists, many partners must work together in complementary ways; see Table 4 for suggested action steps. Here, we give ideas but not explicit directives as it has always been the membership of SPR that takes ownership of activities; consequently, it is the skills, talents, and resources of SPR members that will operationalize these general suggestions.

**Table 4:** Implications for Next Steps to Plan Training Opportunities

Implications	Action steps
Continue to support training: build logical training sequences across a range of topics	• Continue to support training across a broad range of topics • Create and link SPR members to training opportunities in high-interest, specialized topics in order to add value to the field • Build a logical training sequence for specialized topics that include scaffolded content delivered through a range of training modalities • Ensure that scaffolded content can also serve as a standalone training
Continue to hold workshops before and/or after the annual SPR meeting	• Continue to hold in-person preconference workshops • Plan two noncompeting data analysis preconference workshops each year, with at least one having a focus on quantitative data analysis methods and one occasionally focusing on qualitative analyses and/or mixed methods • Include an annual preconference workshop on a preventive intervention topic • Plan a multiyear series of workshops or offer specialization tracks each year, including tracks for research methods, preventive interventions, and teaching • Use the topic-specific results from this report to identify and give preference to/actively encourage submissions related to topics of “special interest”
Add a session at the annual conference focused on teaching	• Include at least one session at the annual meeting dedicated to teaching; this could be a roundtable, networking opportunity, preconference workshop, or other format designed to facilitate the exchange of ideas and the development of teaching skills • Develop a workgroup under the umbrella of the SPR training committee dedicated to strategic planning for these professional development efforts
Coordinate with professional organizations and training institutions to provide professional development opportunities	• Leverage existing resources by coordinating with other professional organizations and training institutions to provide training in areas such as project management, interpersonal and professional skills, and communications • Use SAMHSA’s Prevention Technology Transfer Centers charged with developing training programs for regional and national training needs • Add a list of resources garnered from prevention science graduate programs, postdoctoral fellowships, and centers to the SPR website • Communicate quality high external training opportunities via the SPR listserv • Provide space at the annual meeting for members to connect with policy makers and education and advocacy organizations (e.g., Research America) through events such as a ‘Lunch with the Leaders’
Support self-initiated learning opportunities and tracking mechanisms	• Develop a workgroup within SPR’s training committee to recommend self-initiated learning solutions to the SPR board • Track the use of any self-initiated training opportunities launched following workgroup recommendations and measure participation outcomes
Facilitate high-quality, relevant, ethics training, to become a leader in training on ethics in prevention research	• Provide ethics training in hot-topic areas (e.g., adaptive interventions, big data, machine learning) to increase member interest in ethics trainings • Embed ethics training into existing trainings, courses, and webinars, especially on topics with unique privacy and confidentially considerations • Increase access to trainings and credible resources regarding the privacy and confidentiality implications of emerging digital tools and methods • Use a keynote address to highlight emerging and conference theme relevant ethical issues at the SPR annual meeting
Develop more engaging experiential training opportunities	• Form a workgroup to brainstorm and recommend experiential learning/training, including, but not limited to, internships, experiential coordination to plan an evaluation with a local youth-serving organization, networking events at the annual conference
Target training to specific membership groups	• Continue to award conference and preconference travel scholarships to diverse members, including non-White and early-career members • Include early-career and non-White members in planning activities in order to fill gaps in training needs highlighted by members of these groups • Engage members of the Early Career Preventionist Network and the Diversity Network when planning training efforts • Divide training opportunities into different tracks based on experience, to maximize the relevance of training opportunities to each audience • Use a race impact analysis decision tool (e.g., racial impact analysis) to help make decisions about training (Annie E Casey Foundation, 2016)
Conduct a training needs assessment every 10 years	• Assess training needs every 10 years to capture changing needs and to provide ample time for implementation of recommendations • Use mixed methods to identify training needs to be assessed on the qualitative portion of the training needs assessment • Solicit feedback fsrom members who have not renewed their SPR membership, to learn more about whether specific training opportunities might have prevented members from disaffiliating • Add specific question about engaging with policy makers • Ask respondents to identify the most urgent need
Link the collection of other SPR-specific surveys to the annual meeting to facilitate response rates	This survey had a slightly higher than typical response rate, compared with prior SPR surveys, potentially due to the following reasons: • Administration coincided with the annual meeting • Survey could be answered on any Internet-capable, hand-held device • Multiple outreach and communication attempts were conducted • Improved data collection procedures on Qualtrics, such as identified survey links, enabled respondents to complete the survey across multiple sessions

Nine recommendations specifically related to training and two recommendations related to collecting information emerged: (a) build logical training sequences; (b) continue to hold preconference workshops; (c) add a session at the annual conference related to teaching; (d) coordinate with other professional organizations and training institutions; (e) support self-initiated learning; (f) facilitate relevant ethics training; (g) develop engaging experiential training opportunities; (h) target training to specific groups; (i) regularly assess training needs; and (j) link data collection to annual meetings. This feedback has been reviewed and has already started to be integrated into the training committee’s work and the board’s strategic plan.

### Limitations

Despite the depth and breadth of this study, there are a few limitations. The sample was mostly representative of SPR’s 2017 membership, but it was not randomly selected or intentionally representative. As a result, we cannot rule out selection effects. For example, members with more interest in training, or with a less busy summer schedule, may have been more likely to complete the questionnaire than other members. However, given the variability in interest across topics, it is likely that respondents represented a broad cross section of SPR members and did not pull from one group more than others. Relatedly, although 28% participation is higher than or comparable with similar surveys (Veronneau et al. 2012; Yetter and Capaccioli 2010; Zhang et al. 2019), other procedures may help increase participation.

The subsample composed of people of color was small and included a variety of races and ethnicities. Due to the small sample size, it was not feasible to examine specific races/ethnicities separately as their training needs may be different. The sample sizes for career levels also varied. Although study results meaningfully suggest differences and similarities in these subpopulations, differences in sampling may change rates of endorsement or inference. Consequently, even though these results inform planning and general targeting, they may not be representative of all SPR members or professionals in the broader field of prevention science; these results can be further explored in subpopulation-specific discussions. The sample slightly overrepresented early-career members. Thus, the results can most readily be generalized to the needs of early-career individuals. Because the link for the questionnaire was sent out multiple times, the data could have included multiple responses from the same person.

After much debate, we decided to ask individuals about the likelihood of participating in training, versus feeling that they had enough training in the topic or that the topic was not relevant to them. We selected this set of response options because we wanted to try to assess the likelihood of behavior, not just attitudes. Throughout this article we have equated positive endorsement, a somewhat likely or very likely response, as indicating an interest in training. Conversely, we allowed for two negative responses: one, “this area is not relevant to me” and two, “I have enough training in this area.” It is possible that using a different set of response options could have led to slightly different results. It is possible that the two negative response options did not capture all possible reasons why someone may not be at least somewhat likely to participate in training. However, we also allowed individuals to skip items. Although we still stand by this set of response options as we feel it most strongly links attitudes to behavior, future training needs assessments may want to consider other response options before using this one.

## Conclusion

The study goal was to provide an updated assessment of the training needs of SPR members. These results suggest training opportunities of interest for prevention scientists in addition to training in the foundational areas (Biglan et al. 2011). These results have been shared to the board and have already started to inform training opportunities sponsored by SPR. Findings suggest that members across career stages and demographics are interested in training about a range of prevention science topics. Self-initiated training, webinars, and preconference workshops were the most endorsed modality and early-career individuals and people of color expressed the most interest in training. These findings suggest that the SPR membership is invested in training. As SPR continues to grow, repeated assessment of member needs is critical.

## Electronic Supplementary Material

ESM 1 (DOCX 25 kb)

ESM 2 (DOCX 58 kb)

ESM 3 (DOCX 35 kb)

ESM 4 (DOCX 15 kb)

ESM 5 (DOCX 13 kb)
