# Supplementary material for: The Society for Prevention Research 20 Years Later: a Summary of Training Needs
Source: Prev Sci. 2020 Aug 3;21(7):985–1000. doi: 10.1007/s11121-020-01151-1 (PMC7462903; doi:10.1007/s11121-020-01151-1)
Supplement: Supplementary file 1 — (DOCX 25 kb) [file 11121_2020_1151_MOESM1_ESM.docx]

**KEY INFORMANT INTERVIEWS**

**Full Protocol and Results**

**Methods**

**Participants**

Thirteen participants completed semi-structured key informant interviews. Participants included graduate students (n=2), early career- (n=3), mid-career- (n=4), and senior career-level scientists (n=4). Five key informants identified as male and eight as female. Participants received degrees in Education, Human Development and Family Studies, Psychology, Public Health, and Social Work, and work in the fields of Prevention Science, Psychology, Public Health, and philanthropy. Current work settings included academic (both tenure track and non-tenure track) and non-academic (e.g., funders of prevention research; nonprofit research institutes) settings.

**Procedure**

The Penn State Institutional Review Board approved the study as exempt before participant recruitment began. The SPR Needs Assessment Task Force developed the key informant interview guide across a series of four meetings. We based initial questions on a review of relevant literature, previous training surveys conducted by SPR, and knowledge of the field. We then used an iterative feedback process to refine questions. The final interview guide (see Appendix 1) used a semi-structured format to allow for more in-depth questioning as appropriate. This guide included general questions for all key informants, as well as a few questions specific to graduate students and a few questions specific to those who had already completed their postgraduate degree. The interview guide included five sections: Background, Personal Career Reflection (not given to graduate students), Current Training, Student Training, and Big Picture (two questions were included in this section specific to funders of prevention research).

The Task Force generated a list of possible key informants to represent the different career levels, workplace settings, and demographic characteristics of SPR members. Individual Task Force members contacted one to three key informants each via email, provided them with a brief description of the study and a consent form to review, and scheduled an interview with the key informants who agreed to participate. Only one potential informant declined due to scheduling conflicts. Interviewers used the semi-structured interview guide and probed for further clarification and detail when appropriate. During the interview, interviewers entered all data into Survey Monkey, which had skip patterns to ensure that they only asked key informants questions pertinent to their career level. Interviews lasted approximately one hour and were completed between January 21, 2016 and February 29, 2016.

**Data Analysis**

A subset of Task Force members first coded the contents of the key leader interviews using HyperRESEARCH Researchware, Inc., a free qualitative data analysis software. Each involved Task Force member was assigned a set of similar-themed questions to code using emergent coding techniques. Rather than coding at the level of the person and counting the frequency each person endorsed a particular theme at least once for each question, the ***data was coded at the item level***, compiling all content from a particular item across participants and counting the frequency of times each code was mentioned across all responses, out of the total number of coded items. This technique allowed the Task Force to better understand the significance of themes, as a theme that was mentioned more than once was then coded the appropriate number of times, within each respondent’s answers. Of note, the “n” refers to the total number of codes that emerged from the item’s aggregate content not the number of individuals who mentioned the code.

**Results**

**Personal Career Reflection**

**What areas do you wish you had better training on by the time you completed your degree?** When asked about areas that participants wished they had better training in, several overarching themes emerged out of a total of 72 coded ideas: *Research Methods* (n=26), *Soft Skills* (n=16), *Theoretical Foundations* (n=10), *Programs* (n=9), *Writing* (n=7), and *Teaching/Mentoring* (n=4). For Research Methods, responses included any mention of data analysis or research methods (e.g. evaluation designs, longitudinal models, designs specific to prevention). Participants mentioned several different types of “soft” skills such as methods of community collaborations, time management skills, and navigating employment opportunities/expectations. Theoretical foundations included systems theory, etiologic theory, and theories related to implementation. Related to Programs, participants discussed wanting more training in evidence based programs and the history of prevention. Training in Writing consisted of three components, grant writing, writing for non-academics, and learning the publication process. Finally, for Teaching/Mentoring, participants desired more training in how to teach as well as mentor.

**What skills have benefitted you the most to this point in your career?**  Participants were asked to name up to 5 skills that benefitted them the most, and this resulted in a total of 54 coded ideas. The themes that emerged included: *Soft Skills* (n=20), *Theoretical Foundations* (n=15), *Miscellaneous* (n=7), *Statistics* (n=6), and *Writing* (n=6). For Soft Skills, participants reported benefitting from such things as knowing how to collaborate or work as a team, good mentorship models, and time management skills. Theoretical Foundations included intervention development and logic models, developmental theories, and being able to critically review the literature. Miscellaneous skills included working with children and families, project management, and budget management. Statistics included quantitative, methodological, and qualitative skills. Writing skills included grant writing, writing for non-academics, and how to tell a story.

**Current Training**

**What do you currently need training on?** Participants were also asked about the top five skills or areas they needed training on currently. This resulted in a total of 57 coded ideas which were categorized into six themes: *Theoretical Foundation* (n=13); *Statistics* (n=11); *Soft Skills* (n=9); *Miscellaneous* (n=9); *Writing* (n=8); and *Project Management* (n=7). For Theoretical Foundation, participants mentioned needing skills in research design, Implementation Science, Theory of Change, and intervention/logic models. For Statistics, quantitative skills were most often mentioned, but this theme also included qualitative methods, economic/cost benefit analysis, and power analysis. Soft Skills included how to be forward thinking/visionary, pursuing areas despite major challenges, time management/multitasking, mentorship, and collaboration. Miscellaneous skills included understanding the funding landscape and finding sources of funding beyond NIH, teaching, and working with organizations beyond academia. For Writing, participants wanted to develop better writing skills to tell a story and grant writing. Project Management skills included conflict resolution, staff management, leadership, and meeting facilitation.

**How do you currently fulfill your training needs?** When asked about how participants currently fulfill ongoing training needs a total of 63 coded ideas emerged organized into three overall themes: *Organized Group Training* (n=31), *Solo Training* (n=24), and *Organizations That Hold Trainings* (n=8). Organized Group Training included in-person workshops, webinars, conferences, courses (for current students), and preconference workshops. Solo Training included one-on-one outreach to experts, literature searching, trying out new methods, and online searching. Organizations That Hold Trainings included attending employer trainings and organizations that conduct trainings regularly.

**What areas have you received additional training?** All participants, except current graduate students, were asked since receiving their degree, in what areas have they received additional training. The responses resulted in 33 coded ideas across two general themes: *Miscellaneous* (n=24) and *Methodology* (n=9). Miscellaneous included areas such as specific topic areas, grant writing, business development, and teaching. Methodology included both specific methodologies and more general methodology training.

**Student Training**

**What skills are most important for a prevention science graduate student to learn?** All participants, except current graduate students, were also asked what skills they believed were most important for a prevention science graduate student to learn while in a graduate program. Responses resulted in 59 coded ideas across seven themes: *Research Methods and Evaluation* (n=19)*, Theory* and *Conceptual Frameworks* (n=12)*, Professional Development* (n=11)*, Statistical Methods* (n=11)*, Multidisciplinary and Collaborative Work* (n=4)*, Mentoring* (n=1)*, and Evidence-Based Practice* (n=1).

**What skills does your mentor focus on developing in you?**  Graduate students only were asked, when thinking about their mentor, what skills they believed the mentored focused on during mentorship. Responses resulted in 41 coded ideas across seven themes which were very similar to the themes discussed by the other participants. Themes included: *Manuscript and Grant Writing Activities* (n=11)*; Professional Development Skills* (n=10)*; Research Methods and Evaluation* (n=7)*; Collaborative and Multidisciplinary Work* (n=5)*; Theory* and *Conceptual Framework* (n=4)*; Statistical Methods* (n=3)*;* and *Mentoring* (n=1)*.*

**What skills are important for a new hire or new graduate student?** Non graduate student respondents were asked what skills they looked for in a new hire. Responses to this question resulted in 41 coded ideas. The following eight themes emerged from the 41 coded ideas: *Interpersonal and Collaborative Skills* (n=10)*; Research Design Statistics and Theoretical Background* (n=10)*; Programmatic and Evolving Research* (n=6)*; Productivity* (n=5)*; Thinking Critically and Scientifically Curious* (n=3), *Applied Experience* (n=3); *Fit in Department or Center* (n=2)*; and Teaching Skills* (n=2)*.*

Graduate students were asked the same question from a different perspective, and asked to think about what skills their mentor or supervisor look for in a new graduate student or new hire. A total of 11 coded ideas emerged across eight themes: *Initiative and Autonomy* (n=2)*; Leadership and Ability to Manage Up* (n=2)*; Willingness to Take Direction Learn and Grow* (n=2); *Available for Guidance on Academic Career and Life Decisions* (n=1)*; Aware of Resources and Available Opportunities* (n=1)*; Field Experience* (n=1)*; Research Methods and Statistics* (n=1)*;* and *Time Management* (n=1)*.*

**What courses or experiences would you require in a PhD program in prevention science?** All participants were asked if they were to design a PhD program in prevention science, what courses or experiences would they require of students. This resulted in a total of 64 coded ideas and very similar themes to the question above. The ten themes included: *Research Methods and Statistics* (n=15)*; Applied Experiences* (n=11)*; Theoretical Framework of Prevention Science* (n=10); *Etiology and Epidemiology* (n=5)*; Professional Development* (n=5)*; Developmental Science* (n=4)*; Public Health* (n=4)*; Publications and External Funding* (n=4)*; Teaching and Mentoring* (n=3)*;* and *Collaborative and Multidisciplinary Experiences* (n=3)*.*

**Big Picture**

**What skills do you look for in a new Principal Investigator?** Prevention research funders were asked two additional questions. First, they were ask what skills they look for in the application of a new/first time Principal Investigator of an R01. A total of seven coded ideas emerged across four themes. The themes included: *Match Between Researcher(s)' Abilities and Proposed Project* (n=3); *Writing Skills* (n=2); *Creativity and Innovation* (n=1); and *Knowledge of Literature* (n=1). Second, they were asked what skills, if absent, would cause you to shy away from funding a first time R01 applicant. Only one code/theme emerged which was the *Inability to Make a Logical Argument*.

**What is the most important skill a person needs to be a successful prevention scientist?**  All participants were asked what they believed was the most important skill a person needs to be a productive prevention scientist. A total of 47 ideas were coded and four overall themes emerged. The four themes included: *Research Skills* (n=22)*; Interpersonal Skills* (n=12)*; Personal Qualities* (n=6)*;* and *Work Skills* (n=7)*.* Research Skills included strong knowledge of research methods, quantitative or qualitative statistical skills, core knowledge of prevention science, theoretical knowledge, and knowing the field. Interpersonal Skills included collaboration, engaging with the community, leadership, and communication. Personal Qualities included perseverance and positivity. Work Skills included writing and creativity.

**What is the most important skill a person needs to be an effective prevention science educator or mentor?** Participants were also asked what they thought the most important skill a person needs to be an effective prevention science educator and/or mentor. A total of 33 coded ideas emerged. The same four themes emerged as in the previous question, however, the order differed. For this question, *Interpersonal Skills* (n=16) was the most common code and included desire and commitment to mentor, relationship building, and communication. *Personal Qualities* (n=9) included motivation to educate/mentor/help people early in their career, being a positive role model, patience, and encouraging. *Research Skills* (n=6) included being current with literature and best practices. *Work Skills* (n=2) included setting standards and making skills relevant to those with whom you are working.

**What are fundamental skills versus more time sensitive skills / era-specific skills for Prevention Scientists?** Finally, the last question asked participants if there are some skills or fundamentals that they considered to be core aspects of prevention science, versus some skills that are more time sensitive, or era specific. This question resulted in a total of 62 coded ideas which were categorized into nine themes. The nine themes included: *Fundamental Methods and Design* (n=13); *Fundamental Individual Skills* (n=9); *Fundamental Statistics* (n=9)*; Fundamental Prevention Program Understanding* (n=4)*; Fundamental Theory/Approaches* (n=5); *Hot Topic Skills* (n=11); *Hot Topic Content Area* (n=7)*; Hot Topics Technology* (n=2)*;* and *Miscellaneous* (n=2). Fundamental Methods and Design included skills in methodology and study design. Fundamental Individual Skills included the ability to collaborate/do interdisciplinary work, communication, leadership, innovation, and critical thinking. Fundamental Statistical Skills included statistical skills, mainly focused on quantitative methods. Fundamental Prevention Program Understanding included understanding types of prevention as well as types of intervention programs. Fundamental Theory/Approaches included basic theory as well as systems science. Hot Topic Skills included specific statistics training as methods gain popularity, methods skills as new methods develop, and keeping current on new theory and adapting to new environments. Hot Topic Content Area included learning new content areas and also staying current with evolving knowledge. Hot Topics Technology included staying up to date with technologies for interventions and working with different populations. Miscellaneous included the idea that technology is here to stay and having access to resources to do what you need to do.

**Summary**

Overall, participants repeated the importance of soft skills (i.e., working with a team, time management), statistics, theoretical foundations, and writing had benefited them the most thus far in their careers. However, they also felt that skills in these same areas, as well as research methods, programs, and teaching and mentoring, were still needed. When asked about student training in prevention science many of the same themes emerged such as research methods, theoretical foundations, and statistical methods in addition to collaborative and multidisciplinary work and professional development. When asked about the most important skill for a successful prevention scientist, research skills, interpersonal skills, personal qualities (e.g. perseverance/curiosity), and work skills (e.g. writing/creativity) emerged. Participants reported getting training through group trainings (e.g. workshops, webinars, conferences), solo training (e.g. one-on-one outreach to experts, literature searching, trying out new methods, and online searching), and organizations that conduct regular trainings.

**Key Informant Interview Protocol**

**Overview of the Study**

SPR is dedicated to improving the quality of prevention science in order to promote health and well-being across diverse populations. In order to achieve this goal, it is important to ensure that the organization’s members are well trained in all aspects of prevention science, including methods for conducting etiological research, designing and testing preventive interventions, and ensuring that evidence-based and effective interventions are well implemented and widely disseminated.

In 2011, an SPR task group produced a white paper (http://www.preventionresearch.org/advocacy/#SofK) identifying the core areas of knowledge and training considered necessary for prevention scientists and practitioners. The SPR training committee is currently updating this information and will be surveying the membership about its training needs. This feedback will help us achieve one of the SPR Strategic Goals, to promote education and training opportunities for the current and next generation of prevention scientists.

Prior to surveying the full SPR membership, a training committee Task Force is conducting open-ended interviews with a small group of prevention scientists at various stages of their career. The goal is to help us begin to identify the skills and knowledge that are considered critical to prevention scientists of all backgrounds. We appreciate your willingness to assist in this work.

**Instructions**

During this interview, we will be asking open-ended questions regarding the skills and knowledge you consider to be most important in training new prevention scientists and most beneficial in enhancing the skills of more established prevention scientists. We will also ask you to consider the skills you think have benefitted you the most in your career and to identify particular areas that you would like further training in. When answering these questions, feel free to identify very specific or very broad skills or knowledge that would be useful to prevention science researchers, educators, mentors, developers, evaluators, and/or funders.

**Part 1: Background**

***We’re going to begin by asking a set of questions about your background, education, and work experience.***

Non-grad (in the UK, non-postgraduate) Students

1. In what year was your PhD obtained? (NOTE SPR CAREER DEFINITION)
2. What field was your PhD in?
3. Did you do a postdoc as your first job out?
   - Yes
   - No
4. Where are you in your career (early, mid, senior, something else)? [*main question idea: assess the perspective they bring to the field*]
   - Early
   - Mid
   - Senior
   - Other (specify:___________________)
5. What field are you in now, and what is your current position?
6. Have you had other jobs? Other types of jobs? [*main question idea: inform their experience and perspective…*]
7. What roles do you play in the field of prevention science? For example, some see themselves as a researcher, or teacher, applied evaluation specialist; mentor; advocate; something else? Have these roles changed over time? [*main question idea: get a sense of what perspective they bring to the field*]

Grad Students (for participants in the UK, the equivalent is a postgraduate student)

1. What year are you in your program?
2. When will you obtain your PhD?
3. In what field was your bachelors’ degree?
4. Do you have your masters’ degree?
   - Yes
   - No
   - If yes: In what field was your master’s degree?
5. In what field are you obtaining your PhD?
6. Did you take time off before coming back to school (either before your undergrad or graduate degree)?
   - Yes – I took time off before completing my BS or BA degree
   - Yes – I took time off between completing my BS/BA and beginning my Masters/PhD
   - No

*Follow-up if yes*: What type of trainings or positions did you obtain during this time?

1. What are your professional goals, or what role would you like to play in the field of prevention science? For example, some seek to be a researcher, or teacher, applied evaluation specialist; mentor; advocate; something else? Have these goals changed over time?
2. Are you planning to seek an additional training-focused position before your ultimate career goal (for example, a postdoc, or something else)?
   - Yes
   - No

*Follow-up if yes*: What type of position?

**Part 2: Personal Career Reflection [***skip to Part 3 for grad student (or ‘postgraduate’ respondents***]**

***Next, we would like to know more about the skills and knowledge that you have found beneficial in your career.***

1. Thinking back to the first couple of years after your doctoral degree or postdoc (if applicable), what are 5 skills or areas that you wish you had better training on by the time you completed your degree? These can be general to your position, or specific to prevention science?
2. What skills have benefitted you the most to this point in your career (name up to 5)?

**Part 3: Current Training**

***Next, we are interested in learning more about the training experiences you have had or would like to have to supplement your formal education and training in prevention science.***

1. What are the top 5 skills or areas that you need training on now?
2. How do you currently fulfill your ongoing training needs? Clarifying question, if needed: what sources do you rely on when you want to learn about something new or learn about a new area (e.g., webinars, books, online resources, talk with experts, attend trainings, etc.)?
3. *Non-grad student respondents*: Since receiving your degree, in what areas have you received additional training?
4. *Grad student (postgraduate) respondents*: While in your doctoral program, have you received training outside of your program (e.g., additional statistical trainings, off-site training visits, federally-funded opportunities, etc.)?

- Yes
- No

*If yes*: Please describe them:

**Part 4a: Student Training**

***Now, thinking more broadly, we will be asking you about the areas of knowledge you think should be part of prevention science training for students as well as more established prevention scientists*.**

1. What skills do you think are most important for a prevention science graduate student to learn in graduate school?
2. *Non-grad student (non postgraduate) respondents*: If applicable, what skills do you focus on training within your own graduate students (or others that you manage)?
3. *Grad student (postgraduate) respondents*: Thinking of your mentor, what skills do they focus on when mentoring you?
4. *Non-grad student (non postgraduate) respondents*: If applicable, what skills do you look for in a new hire (note the type of position the respondent is thinking of when they respond)?
5. *Grad student (postgraduate) respondents*: Thinking of your mentor or supervisor, what skills do they look for in a new graduate student or new hire?
6. If you were to design a PhD program in prevention science, what courses or experiences would you require of students?

**Part 4b: Big Picture**

1. *NIH Project Officer respondents*: What skills do you look for in the application of a new first time Principal Investigator of an R01?
2. *NIH Project Officer respondents*: What skills, if absent, would cause you to shy away from funding a first time R01 applicant?
3. What is the most important skill that a person needs to be a productive prevention scientist? [Then probe with second most important, and then probe for “any other skills?”, so that we can get a sense of ranking – they can name up to 5]
4. What is the most important skill a person needs to be an effective prevention science educator and/or mentor? [Then probe with second most important, and then probe for “any other skills?”, so that we can get a sense of ranking – they can name up to 5]
5. Are there some skills or fundamentals that you consider to be core aspects of prevention science, versus some skills that are more time sensitive, or era specific? For example, regression analysis may be an important fundamental that withstands the test of time, but something like latent class analysis may be more time-specific. What are they (specify time sensitive, era-specific vs. fundamental)?
