# Supplementary material for: The Society for Prevention Research 20 Years Later: a Summary of Training Needs
Source: Prev Sci. 2020 Aug 3;21(7):985–1000. doi: 10.1007/s11121-020-01151-1 (PMC7462903; doi:10.1007/s11121-020-01151-1)
Supplement: Supplementary file 2 — (DOCX 58 kb) [file 11121_2020_1151_MOESM2_ESM.docx]

**Thank you for agreeing to participate in this survey! This 20-30 minute survey assesses the training needs of professionals in the field at all levels: early career, mid-career, and senior. It covers the knowledge and skills that prevention researchers and educators often need to conduct cutting-edge research and pass along their related knowledge. Your participation is extremely valuable, because the Society for Prevention Research (SPR) will use these results to plan training opportunities for the next 10 years. Ensuring appropriate and high quality training opportunities is very important to SPR. SPR expects that the provision of training will increase the prevention science skills of its members and enhance the field of prevention science.**

**Please note, you may find some areas less relevant to your experiences, but as a whole all areas are important for the field of Prevention Science. Therefore, when answering these questions, please think beyond the current training available to you and consider all of your training needs. Also, please consider that training could take many forms, from self-initiated learning (e.g., reading materials, or watching presentations on your own), to one-on-one mentoring opportunities, Webinars, preconference or other workshops, or even an experiential learning opportunity.**

**Section 1: Theory**

**Let’s start off with some questions about the theories that guide the development of preventive interventions.**

**Given the opportunity, how likely are you to participate in training in the following areas?**

|  | Not at all – I already have adequate knowledge or skills in this area | Not at all – this area is not relevant to me | Somewhat likely | Very likely |
| --- | --- | --- | --- | --- |
| 1. The etiology / epidemiology of health behaviors, including key conceptual models |  |  |  |  |
| 1. Human and child developmental theory |  |  |  |  |
| 1. Key ideas and principles in public health |  |  |  |  |
| 1. The history and conceptual foundations of prevention science, including the prevention research cycle |  |  |  |  |
| 1. Theories of change and related conceptual frameworks |  |  |  |  |
| 1. Complex systems and system theory |  |  |  |  |
| 1. The role of context in shaping health behaviors and interventions |  |  |  |  |
| 1. Theories regarding the mechanisms for addressing health disparities |  |  |  |  |

1. **[IF ‘somewhat’ or ‘very likely’ endorsed for any of the prior responses…] What type of training event(s) or modality(ies) would be the most useful for developing your knowledge/skills in the theories that guide the development of preventive interventions? *Select all that apply***

- Self-initiated learning (e.g., reading materials, or watching presentations on your own)
- One-on-one mentoring/coaching/consulting
- Webinar
- SPR Preconference Workshop
- One-day in-person workshop (not SPR conference affiliated)
- Multi-day in-person workshop (not SPR conference affiliated)
- Experiential education (e.g., internship, short-term hands-on experience on a project, or a client)

**Section 2: Preventive Interventions**

**Now let’s move on to questions about developing, evaluating, and implementing preventive interventions (i.e., practices, policies, and programs), as well as methods for increasing the large-scale dissemination and sustainability of these interventions.**

**Given the opportunity, how likely are you to participate in training in the following areas?**

|  | Not at all – I already have adequate knowledge or skills in this area | Not at all – this area is not relevant to me | Somewhat likely | Very likely |
| --- | --- | --- | --- | --- |
| 1. Ability to identify effective (i.e., evidence-based) and ineffective preventive interventions |  |  |  |  |
| 1. Developing intervention logic models to specify the theory of change, including the risk and protective factors and short- and long-term outcomes targeted by the intervention |  |  |  |  |
| 1. Creating materials, manuals, protocols, and guidelines for intervention delivery |  |  |  |  |
| 1. Selecting and training intervention facilitators, and creating an appropriate model of technical assistance |  |  |  |  |
| 1. Recruiting, engaging, and retaining participants |  |  |  |  |
| 1. Soliciting community input and collaboration in the development, testing, and/or implementation of interventions (i.e., community based participatory research) |  |  |  |  |
| 1. Consideration of cultural competency in designing, delivering, and adapting interventions |  |  |  |  |
| 1. Targeting prevention interventions to reduce health disparities |  |  |  |  |
| 1. Implementation research (e.g. evaluating implementation fidelity and adaptation) |  |  |  |  |
| 1. Dissemination research (e.g., methods for increasing the adoption and large-scale use of evidence-based interventions) |  |  |  |  |
| 1. Incorporating new technologies (e.g., cell phones, social media, etc.) into the design, implementation, and/or evaluation of interventions |  |  |  |  |
| 1. Understanding the unintended negative effects of interventions |  |  |  |  |

1. **[IF ‘somewhat’ or ‘very likely’ endorsed for any of the prior responses…] What type of training event(s) or modality(ies) would be the most useful for developing your knowledge/skills in developing, evaluating, and implementing preventive interventions? *Select all that apply***

- Self-initiated learning (e.g., reading materials, or watching presentations on your own)
- One-on-one mentoring/coaching/consulting
- Webinar
- SPR Preconference Workshop
- One-day in-person workshop (not SPR conference affiliated)
- Multi-day in-person workshop (not SPR conference affiliated)
- Experiential education (e.g., internship, short-term hands-on experience on a project, or a client)

**Section 3: Research Methods, Design, and Evaluation**

**Now let’s move on to questions more specific to research design and evaluation methods.**

**Given the opportunity, how likely are you to participate in training in the following areas?**

|  | Not at all – I already have adequate knowledge or skills in this area | Not at all – this area is not relevant to me | Somewhat likely | Very likely | |
| --- | --- | --- | --- | --- | --- |
| 1. Experimental design (e.g., group/cluster randomized controlled trials) |  |  |  |  | |
| 1. Non-experimental and quasi-experimental design (e.g., regression discontinuity, propensity scoring) |  |  |  |  | |
| 1. Hybrid designs combining effectiveness and implementation |  |  |  |  | |
| 1. Mixed- or multi-method hybrid qualitative / quantitative research design |  |  |  |  | |
| 1. Longitudinal design (e.g., cohort sequential, time series, ecological momentary assessment) |  |  |  |  | |
| 1. Adaptive intervention design (i.e., different dosages of certain prevention or treatment components are assigned to different individuals, and/or within individuals across time, with dosage varying in response to the intervention needs of individuals) |  |  |  |  |  |
| 1. Survey sampling methods (i.e., the process of selecting a sample of elements from a target population to conduct a data collection effort) |  |  |  |  | |
| 1. Biological and physical data collection and analysis (e.g., genetic assessment, psychophysiology, fMRI) |  |  |  |  | |
| 1. Data collection and survey design with non-native English speakers / non-literate populations |  |  |  |  | |
| 1. Data management (e.g., tracking, cleaning, storing, combining multisite, longitudinal datasets) |  |  |  |  | |
| 1. Ethical practices in research (e.g., human subjects protections such as: beneficence & non-malfeasance, fidelity and responsibility, integrity, justice, respect for people’s rights and dignity) |  |  |  |  | |

1. **[IF ‘somewhat’ or ‘very likely’ endorsed for any of the prior responses…] What type of training event(s) or modality(ies) would be the most useful for developing your knowledge/skills in research design and evaluation methods? *Select all that apply***

- Self-initiated learning (e.g., reading materials, or watching presentations on your own)
- One-on-one mentoring/coaching/consulting
- Webinar
- SPR Preconference Workshop
- One-day in-person workshop (not SPR conference affiliated)
- Multi-day in-person workshop (not SPR conference affiliated)
- Experiential education (e.g., internship, short-term hands-on experience on a project, or a client)

**Section 4: Data Analysis**

**Now let’s move on to questions more specific to data analysis strategies. We will first ask you how likely you are to attend training in quantitative and qualitative data analysis. If you indicate that you are likely to pursue training in one or more of these areas, you will then be asked about specific analytical methods in which you would like to be trained.**

**Given the opportunity, how likely are you to participate in training in…?**

1. **Quantitative Analytic Methods**

- Not at all – I already have adequate knowledge or skills in this area [*skip quantitative analytic follow-up items*]
- Not at all – this area is not relevant to me [skip *quantitative analytic follow-up items*]
- Somewhat likely
- Very likely

[Follow-up if “somewhat” or “very” was selected]

**You indicated that you would be somewhat or very likely to participate in training for quantitative analytic methods. Now we would like to know more about the particular topics you need training in. For which of the following topics are you likely to attend training? Please read through the list and select all that apply (first column). You can also endorse whether a particular statistical method is unknown to you (second column). Please note, not every item requires a response – only select those that you would like training in or where the statistical method is unknown to you.**

|  | Would likely attend training | Statistical method unknown |
| --- | --- | --- |
| 1. Agent Based Modeling |  |  |
| 1. Analysis of High-Dimensional Data |  |  |
| 1. Analysis of Small Sample Data |  |  |
| 1. Bayesian Methods |  |  |
| 1. Complier Average Casual Effect (CACE) Analysis |  |  |
| 1. Causal Inference |  |  |
| 1. Cost-Effectiveness Methods |  |  |
| 1. Data Mining |  |  |
| 1. Decision Analysis |  |  |
| 1. Econometric Methods |  |  |
| 1. General Linear Modeling (including regression, multivariate analysis) |  |  |
| 1. Generalized Linear Modeling (Logistic, Poisson, Gamma, etc.) |  |  |
| 1. Genome-Wide Statistical Analysis |  |  |
| 1. Geospatial Analysis |  |  |
| 1. Growth Modeling |  |  |
| 1. Individual Person-Level Meta-Analysis |  |  |
| 1. Integrative Data Analysis |  |  |
| 1. Item Response Theory |  |  |
| 1. Latent Class and Latent Variable Modeling |  |  |
| 1. Measurement Theory and Methods (EFA, CFA, including survey design and scale development) |  |  |
| 1. Mediation Analysis |  |  |
| 1. Meta-Analysis (of summary statistics) |  |  |
| 1. Methods for Analysis of Intensive or Long Longitudinal Data |  |  |
| 1. Microsimulation Methods |  |  |
| 1. Missing Data Methods (multiple imputation, full information maximum likelihood) |  |  |
| 1. Mixture Models (including growth and regression mixture models, longitudinal data, or data collected at multiple levels, “nested” or “clustered” data) |  |  |
| 1. Moderation Analysis |  |  |
| 1. Multi-Level or Hierarchical Regression |  |  |
| 1. N of 1 Experiments |  |  |
| 1. Network Analysis |  |  |
| 1. Non-parametric Statistical Methods |  |  |
| 1. Propensity Score Methods |  |  |
| 1. Psychometric Methods |  |  |
| 1. Simulation Methods |  |  |
| 1. Statistical Power Analysis |  |  |
| 1. Structural Equation Models |  |  |
| 1. Subgroup Analysis |  |  |
| 1. Survey Data Analysis |  |  |
| 1. Survival Analysis / Event History Analysis (i.e., analysis of the expected duration of time until a particular event or set of events happens) |  |  |
| 1. System Dynamics |  |  |
| 1. Systems Engineering Methods |  |  |

**Given the opportunity, how likely are you to participate in training in…?**

1. **Qualitative Analytic Methods**

- Not at all – I already have adequate knowledge or skills in this area [*skip qualitative analytic follow-up items*]
- Not at all – this area is not relevant to me [skip *qualitative analytic follow-up items*]
- Somewhat likely
- Very likely

**[Follow-up if “somewhat” or “very” was selected]**

**You indicated that you would be somewhat or very likely to participate in training for qualitative analytic methods. Now we would like to know more about the particular topics you need training in. For which of the following topics are you likely to attend training? Please read through the list and select all that apply (first column). You can also endorse whether a particular statistical method is unknown to you (second column). Please note, not every item requires a response – only select those that you would like training in or where the qualitative analysis method is unknown to you.**

|  | Would likely attend training | Qualitative analysis method unknown |
| --- | --- | --- |
| 1. Alternative/Authentic Assessment |  |  |
| 1. Case Studies |  |  |
| 1. Content Analysis |  |  |
| 1. Document Analysis |  |  |
| 1. Focus Groups |  |  |
| 1. Key Informant Interviews |  |  |
| 1. Structured Observation |  |  |

1. **[IF ‘somewhat’ or ‘very likely’ endorsed for any of the prior responses…] What type of training event(s) or modality(ies) would be the most useful for developing your knowledge/skills in data analysis techniques? *Select all that apply***

- Self-initiated learning (e.g., reading materials, or watching presentations on your own)
- One-on-one mentoring/coaching/consulting
- Webinar
- SPR Preconference Workshop
- One-day in-person workshop (not SPR conference affiliated)
- Multi-day in-person workshop (not SPR conference affiliated)
- Experiential education (e.g., internship, short-term hands-on experience on a project, or a client)

**Section 5: Teaching and Mentoring**

**Many prevention scientists also mentor and teach students. Below, we ask about training you might need to be a more effective mentor and/or teacher.**

1. **We’ll start with mentoring. Do you…**

- Currently mentor now
- Expect to mentor in your future
- Not currently mentor and do not expect to ever need training in this area [*skip entire mentoring section*]

**Given the opportunity, how likely are you to participate in training to help you mentor students or other early career individuals in the following areas?**

|  | Not at all – I already have adequate knowledge or skills in this area | Not at all – this area is not relevant to me | Somewhat likely | Very likely |
| --- | --- | --- | --- | --- |
| 1. Establishing a mentoring relationship |  |  |  |  |
| 1. Giving constructive criticism and feedback |  |  |  |  |
| 1. Successfully guiding students through the undergraduate or graduate thesis or PhD dissertation process |  |  |  |  |
| 1. Applying for external grant funding |  |  |  |  |
| 1. Collaborating as part of a team (e.g., project management, multidisciplinary collaboration, interpersonal skills) |  |  |  |  |
| 1. Job search and interview skills for both academic and non-academic positions |  |  |  |  |

1. **[IF ‘somewhat’ or ‘very likely’ endorsed for any of the prior responses…] What type of training event(s) or modality(ies) would be the most useful for developing your knowledge/skills in mentoring? *Select all that apply***

- Self-initiated learning (e.g., reading materials, or watching presentations on your own)
- One-on-one mentoring/coaching/consulting
- Webinar
- SPR Preconference Workshop
- One-day in-person workshop (not SPR conference affiliated)
- Multi-day in-person workshop (not SPR conference affiliated)
- Experiential education (e.g., internship, short-term hands-on experience on a project, or a client)

**Now a few questions specific to teaching.**

1. **Do you teach in your current position, or do you expect to teach in a position in your future? *Please select the best response***

- Currently teach now
- Expect to teach in your future
- Not currently teach and do not expect to ever need training in this area [*skip entire teaching section*]

**Given the opportunity, how likely are you to participate in training with regards to teaching courses in the following areas?**

|  | Not at all – I already have adequate knowledge or skills in this area | Not at all – this area is not relevant to me | Somewhat likely | Very likely |
| --- | --- | --- | --- | --- |
| 1. Developing course materials (e.g., constructing a syllabus, identifying readings, balancing breadth and depth of topics) |  |  |  |  |
| 1. Increasing student engagement (including skills for small vs. large classrooms, identifying and engaging students at risk for dropping out) |  |  |  |  |
| 1. Assessment methods (e.g., exam construction, portfolios, papers, formative assessment, grading efficiently, effectively, and reliably) |  |  |  |  |
| 1. Active learning strategies (e.g., flipped classrooms, using clickers, leading discussions, problem-based learning, cooperative learning groups) |  |  |  |  |
| 1. Strategies for teaching online courses |  |  |  |  |
| 1. Skills for discussing “hot button issues” (e.g., prejudice) |  |  |  |  |
| 1. Effective use of teaching assistants (i.e., TAs) |  |  |  |  |

1. **[IF ‘somewhat’ or ‘very likely’ endorsed for any of the prior responses…] What type of training event(s) or modality(ies) would be the most useful for developing your knowledge/skills in teaching? *Select all that apply***

- Self-initiated learning (e.g., reading materials, or watching presentations on your own)
- One-on-one mentoring/coaching/consulting
- Webinar
- SPR Preconference Workshop
- One-day in-person workshop (not SPR conference affiliated)
- Multi-day in-person workshop (not SPR conference affiliated)
- Experiential education (e.g., internship, short-term hands-on experience on a project, or a client)

**Section 6: Practical & Interpersonal Skills**

**Now let’s move on to questions related to practical personal and interpersonal skills. Prevention scientists often work in teams in order to improve the efficiency and efficacy of their work, and success is likely to be related to the degree to which team members have interpersonal skills and experience collaborating with others. Prevention scientists also often juggle many different competing responsibilities that require different skill sets. This section indexes your interest in attending trainings on these topics.**

**Given the opportunity, how likely are you to participate in training in the following areas?**

|  | Not at all – I already have adequate knowledge or skills in this area | Not at all – this area is not relevant to me | Somewhat likely | Very likely |
| --- | --- | --- | --- | --- |
| 1. Developing and maintaining effective collaborative relationships with co-authors and co-investigators (e.g., team building, negotiation, and conflict management skills) |  |  |  |  |
| 1. Initiating collaboration across disciplines (e.g., multidisciplinary collaborations) |  |  |  |  |
| 1. Initiating collaboration with the community (e.g., working relationships with key stakeholders in the community, community prevention practitioners, and constituents) |  |  |  |  |
| 1. Working with others that have different backgrounds and experiences; maintaining diverse teams |  |  |  |  |
| 1. Networking or building/maintaining connections in the prevention science community |  |  |  |  |
| 1. Time management (e.g., managing multiple tasks simultaneously, establishing priorities, flexibility, organization) |  |  |  |  |
| 1. Maintaining motivation and focus when faced with obstacles |  |  |  |  |
| 1. Receiving constructive feedback |  |  |  |  |
| 1. Stress management and maintaining a work-life balance |  |  |  |  |

1. **[IF ‘somewhat’ or ‘very likely’ endorsed for any of the prior responses…] What type of training event(s) or modality(ies) would be the most useful for developing your knowledge/skills in practical personal and interpersonal skills? *Select all that apply***

- Self-initiated learning (e.g., reading materials, or watching presentations on your own)
- One-on-one mentoring/coaching/consulting
- Webinar
- SPR Preconference Workshop
- One-day in-person workshop (not SPR conference affiliated)
- Multi-day in-person workshop (not SPR conference affiliated)
- Experiential education (e.g., internship, short-term hands-on experience on a project, or a client)

**Section 7: Communication**

**Next, we will consider communication skills. There are many modes and mechanisms we use to communicate with our colleagues, funders, community members, and the larger public. This section addresses your interest in attending trainings in these areas.**

**Given the opportunity, how likely are you to participate in training in the following areas?**

|  | Not at all – I already have adequate knowledge or skills in this area | Not at all – this area is not relevant to me | Somewhat likely | Very likely |
| --- | --- | --- | --- | --- |
| 1. Writing manuscripts for peer reviewed journals |  |  |  |  |
| 1. Communicating with foundations |  |  |  |  |
| 1. Communicating with government officials (e.g., National Institutes of Health [NIH]/National Science Foundation [NSF]/Center for Disease Control and Prevention [CDC] project officers) |  |  |  |  |
| 1. Presenting at professional conferences |  |  |  |  |
| 1. Communicating research findings to lay audiences (e.g., policy makers, elected officials, school boards, community members) |  |  |  |  |
| 1. Communicating your work to *the general public* via various social media platforms (e.g., Facebook, Twitter, etc.) |  |  |  |  |
| 1. Communicating your work *to peers, colleagues, or funders* via various social media platforms (e.g., ResearchGate, GoogleScholar Profile, ORCID, LinkedIn, Twitter, Facebook, etc.) |  |  |  |  |

1. **[IF ‘somewhat’ or ‘very likely’ endorsed for any of the prior responses…] What type of training event(s) or modality(ies) would be the most useful for developing your knowledge/skills in communication? *Select all that apply***

- Self-initiated learning (e.g., reading materials, or watching presentations on your own)
- One-on-one mentoring/coaching/consulting
- Webinar
- SPR Preconference Workshop
- One-day in-person workshop (not SPR conference affiliated)
- Multi-day in-person workshop (not SPR conference affiliated)
- Experiential education (e.g., internship, short-term hands-on experience on a project, or a client)

**Section 8: Project Management**

**Next, let’s think about the knowledge and skills it takes to manage a prevention research or evaluation project.**

**Given the opportunity, how likely are you to participate in training in the following areas?**

|  | Not at all – I already have adequate knowledge or skills in this area | Not at all – this area is not relevant to me | Somewhat likely | Very likely |
| --- | --- | --- | --- | --- |
| 1. Effective leadership (e.g., building a research team, delegating responsibilities, supervising and managing the research team, conflict management) |  |  |  |  |
| 1. Understanding the possible funding opportunities for your work (e.g., available grants and contracts for your area of research/evaluation) |  |  |  |  |
| 1. Understanding contract/grant requirements |  |  |  |  |
| 1. Developing work plans, timelines, and expectations; managing schedules, deliverables, and quality |  |  |  |  |
| 1. Developing and tracking budgets and controlling costs; managing budget risks |  |  |  |  |
| 1. Recruiting, hiring, managing, and mentoring project staff |  |  |  |  |
| 1. Meeting management (e.g., agenda development, facilitation, clear action items) |  |  |  |  |
| 1. Evaluating project outcomes (e.g., developing metrics for progress and success and ways to display those metrics, such as “dashboards”) |  |  |  |  |

1. **[IF ‘somewhat’ or ‘very likely’ endorsed for any of the prior responses…] What type of training event(s) or modality(ies) would be the most useful for developing your knowledge/skills to manage a prevention research or evaluation project? *Select all that apply***

- Self-initiated learning (e.g., reading materials, or watching presentations on your own)
- One-on-one mentoring/coaching/consulting
- Webinar
- SPR Preconference Workshop
- One-day in-person workshop (not SPR conference affiliated)
- Multi-day in-person workshop (not SPR conference affiliated)
- Experiential education (e.g., internship, short-term hands-on experience on a project, or a client)

1. **We have just asked you to respond to many items about important topics in Prevention Science. What other insight or perspective can you share to help us understand your training needs and the likelihood that you will take advantage of future training opportunities organized by the Society for Prevention Research?**

________________________________________________________________________________________________________________________________________________________________________________________________________________________________________________________________________________________________________________________________________________________________________________________________________

**Thank you for your participation, we just now have a few background questions.**

1. **What is your current career level?**

- Undergraduate Student
- Graduate Student
- Post-Doctoral Fellow
- First Year Professional
- Early Career
- Mid-Career
- Senior
- Other: ___________

[Follow-up if “other” was selected]

1. **What is the highest degree you have completed? (select all that apply)**

- PhD
- MD
- ScD
- DrPH
- EdD
- JD
- MS
- MA
- MSW
- MPH
- MEd
- MPP
- MPA
- BA/BS
- High School Diploma or GED
- Other: ___________

[Follow-up if “other” was selected]

1. **Gender:**

- Male
- Female
- Prefer Not to Answer

1. **Disabled:**

- Yes
- No

1. **Ethnicity: (*select all that apply*)**

- American Indian, Native American
- Asian, Pacific Islander
- Black, African American
- Spanish, Hispanic, Latino
- White, Caucasian, European
- Other: ___________

[Follow-up if “other” was selected]

1. **What type of organization do you currently work for?**

- College or university
- For-profit organization
- Government agency or organization
- Non-profit research center not affiliated with a university
- Non-profit service agency
- Private practice/consultant
- Other: ___________

[Follow-up if “other” was selected]

**Please enter your email address if you would like to participate in the raffle for the participation prizes.**

Email: ___________

**Thank you!!!**
