# Supplementary material for: The Society for Prevention Research 20 Years Later: a Summary of Training Needs
Source: Prev Sci. 2020 Aug 3;21(7):985–1000. doi: 10.1007/s11121-020-01151-1 (PMC7462903; doi:10.1007/s11121-020-01151-1)
Supplement: Supplementary file 3 — (DOCX 35 kb) [file 11121_2020_1151_MOESM3_ESM.docx]

**Supplementary Material**

Percentages of respondents endorsing “very likely” to participate in training across all eight survey sections

| **Prevention Science Theory Items – Career Level** | **Early**  ***n* = 184** | | **Very Likely**  **Mid**  ***n* = 68** | **Senior**  ***n* = 52** |  |
| --- | --- | --- | --- | --- | --- |
| Mechanisms for addressing disparities | 41% | | 29% | 31% |  |
| Context shaping health behavior | 37% | | 25% | 19% |  |
| Complex systems and systems theory | 29% | | 19% | 23% |  |
| Theories of change | 25% | | 12% | 21% |  |
| Key principles in public health | 24% | | 22% | 17% |  |
| Etiology/epidemiology of health behaviors | 18% | | 22% | 10% |  |
| Foundations of prevention science | 21% | | 12% | 14% |  |
| Human developmental theory | 25% | | 8% | 15% |  |
|  | **Very Likely** | | |  |  |
| **Prevention Science Theory Items – White vs. People of Color** | | **White**  ***n* = 218** | **People of Color**  ***n* = 84** |  | |
| Mechanisms for addressing disparities | 36% | | 42% | -- |  |
| Context shaping health behavior | 31% | | 35% | -- |  |
| Complex systems and systems theory | 27% | | 23% | -- |  |
| Theories of change | 21% | | 25% | -- |  |
| Key principles in public health | 18% | | 33% | -- |  |
| Etiology/epidemiology of health behaviors | 17% | | 23% | -- |  |
| Foundations of prevention science | 17% | | 24% | -- |  |
| Human developmental theory | 17% | | 25% | -- |  |

***Notes***: The n listed for each demographic group is the maximum number of respondents (of that type) who responded to any one topic in this section.

| **Preventive Intervention Topics – Career Level** | **Early**  ***n* = 184** | **Very Likely**  **Mid**  ***n* = 68** | **Senior**  ***n* = 52** |
| --- | --- | --- | --- |
| Incorporating new technologies | 49% | 49% | 49% |
| Consideration of cultural competency | 41% | 21% | 20% |
| Understanding the negative effects of interventions | 31% | 24% | 23% |
| Dissemination research | 42% | 31% | 31% |
| Implementation research | 42% | 30% | 25% |
| Targeting prevention interventions to reduce health disparities | 41% | 30% | 26% |
| Community input and collaboration | 42% | 24% | 14% |
| Recruiting, engaging, and retaining participants | 26% | 18% | 10% |
| Creating materials and guidelines for intervention delivery | 28% | 15% | 6% |
| Developing intervention logic models | 30% | 19% | 10% |
| Training and technical assistance | 25% | 15% | 8% |
| Effective and ineffective interventions | 31% | 18% | 17% |
|  | **Very Likely** | |  |
| **Preventive Intervention Topics – White vs. People of Color** | **White**  ***n* = 217** | **People of Color**  ***n* = 82** |  |
| Incorporating new technologies | 47% | 54% | -- |
| Consideration of cultural competency | 29% | 44% | -- |
| Understanding the negative effects of interventions | 28% | 31% | -- |
| Dissemination research | 34% | 48% | -- |
| Implementation research | 32% | 47% | -- |
| Targeting prevention interventions to reduce health disparities | 32% | 49% | -- |
| Community input and collaboration | 29% | 45% | -- |
| Recruiting, engaging, and retaining participants | 18% | 31% | -- |
| Creating materials and guidelines for intervention delivery | 18% | 31% | -- |
| Developing intervention logic models | 20% | 34% | -- |
| Training and technical assistance | 18% | 27% | -- |
| Effective and ineffective interventions | 20% | 40% | -- |

***Notes***: The n listed for each demographic group is the maximum number of respondents (of that type) who responded to any one topic in this section.

| **Research Methods, Design, Evaluation Topics – Career Level** | **Early**  ***n* = 182** | **Very Likely Mid**  ***n* = 68** | **Senior**  ***n* = 52** |
| --- | --- | --- | --- |
| Hybrid Designs Combining Effectiveness & Implementation | 46% | 31% | 31% |
| Adaptive Intervention Design | 46% | 31% | 26% |
| Mixed or Multi-Method Hybrid Qualitative/Quantitative | 46% | 34% | 15% |
| Longitudinal Design | 47% | 28% | 20% |
| Non-Experimental Design/Quasi-Experimental Design | 45% | 24% | 17% |
| Survey Sampling Methods | 28% | 24% | 8% |
| Experimental Design | 34% | 12% | 14% |
| Data Management | 30% | 22% | 8% |
| Data Collection & Survey; Non-Native English Speakers/Non-Literate Populations | 20% | 12% | 12% |
| Biological & Physical Data Collection & Analysis | 20% | 15% | 14% |
| Ethical Practices | 11% | 13% | 10% |
|  | **Very Likely** | |  |
| **Research Methods, Design, Evaluation Topics – White vs. People of Color** | **White**  ***n* = 217** | **People of Color**  ***n* = 84** |  |
| Hybrid Designs Combining Effectiveness & Implementation | 37% | 47% | -- |
| Adaptive Intervention Design | 36% | 46% | -- |
| Mixed or Multi-Method Hybrid Qualitative/Quantitative | 34% | 49% | -- |
| Longitudinal Design | 35% | 47% | -- |
| Non-Experimental Design/Quasi-Experimental Design | 32% | 43% | -- |
| Survey Sampling Methods | 23% | 32% | -- |
| Experimental Design | 22% | 37% | -- |
| Data Management | 20% | 35% | -- |
| Data Collection & Survey; Non-Native English Speakers/Non-Literate Populations | 14% | 25% | -- |
| Biological & Physical Data Collection & Analysis | 17% | 22% | -- |
| Ethical Practices | 10% | 17% | -- |

***Notes***: The n listed for each demographic group is the maximum number of respondents (of that type) who responded to any one topic in this section.

| **Mentoring Topics – Career Level** | **Early**  ***n* = 154** | **Very Likely**  **Mid**  ***n* = 54** | **Senior**  ***n* = 44** |
| --- | --- | --- | --- |
| Giving Constructive Criticism and Feedback | 37% | 15% | 5% |
| Applying for External Grant Funding | 49% | 11% | 9% |
| Successfully Guiding Students through the Undergraduate or Graduate Thesis or PhD Dissertation Process | 39% | 15% | 7% |
| Job-search and Interview Skills for both Academic and Non-academic Positions | 37% | 9% | 2% |
| Collaborating as Part of a Team | 31% | 9% | 2% |
| Establishing a Mentoring Relationship | 23% | 6% | 2% |
|  | **Very Likely** | |  |
| **Mentoring Topics – White vs. People of Color** | **White**  ***n* = 186** | **People of Color**  ***n* = 64** |  |
| Giving Constructive Criticism and Feedback | 23% | 39% | -- |
| Applying for External Grant Funding | 30% | 45% | -- |
| Successfully Guiding Students through the Undergraduate or Graduate Thesis or PhD Dissertation Process | 25% | 38% | -- |
| Job-search and Interview Skills for both Academic and Non-academic Positions | 22% | 36% | -- |
| Collaborating as Part of a Team | 17% | 36% | -- |
| Establishing a Mentoring Relationship | 13% | 25% | -- |

***Note***: The n listed for each demographic group is the maximum number of respondents (of that type) who responded to any one topic in this section.

| **Teaching Topics – Career Level** | **Early**  ***n* = 140** | **Very Likely Mid**  ***n* = 43** | **Senior**  ***n* = 28** |
| --- | --- | --- | --- |
| Active Learning Strategies | 40% | 26% | 14% |
| Skills for Discussing Hot Button Issues | 44% | 20% | 11% |
| Increasing Student Engagement | 37% | 23% | 10% |
| Assessment Methods | 28% | 19% | 7% |
| Strategies for Teaching Online Courses | 31% | 19% | 14% |
| Effective use of Teaching Assistants | 24% | 10% | 0% |
| Developing Course Materials | 26% | 19% | 3% |
|  | **Very Likely** | |  |
| **Teaching Topics – White vs. People of Color** | **White**  ***n* = 155** | **People of Color**  ***n* = 53** |  |
| Active Learning Strategies | 29% | 49% | -- |
| Skills for Discussing Hot Button Issues | 30% | 49% | -- |
| Increasing Student Engagement | 28% | 40% | -- |
| Assessment Methods | 21% | 33% | -- |
| Strategies for Teaching Online Courses | 23% | 36% | -- |
| Effective use of Teaching Assistants | 16% | 27% | -- |
| Developing Course Materials | 18% | 34% | -- |

***Note***: The n listed for each demographic group is the maximum number of respondents (of that type) who responded to any one topic in this section.

| **Practical and Interpersonal Skill Topics – Career Level** | **Early**  ***n =* 181** | **Very Likely**  **Mid**  ***n =* 67** | **Senior**  ***n =* 49** |
| --- | --- | --- | --- |
| Networking in Prevention Science | 30% | 19% | 10% |
| Initiating Community Collaborations | 46% | 16% | 10% |
| Initiating Interdisciplinary Collaborations | 28% | 21% | 10% |
| Stress Management, Work-Life Balance | 29% | 24% | 9% |
| Time Management | 26% | 21% | 6% |
| Working with Others with Different Backgrounds; Maintaining Diverse Teams | 22% | 13% | 8% |
| Maintaining Motivation with Obstacles | 27% | 16% | 4% |
| Developing and Maintaining Collaborations | 21% | 12% | 10% |
| Receiving Constructive Feedback | 22% | 12% | 6 |
|  | **Very Likely** | |  |
| **Practical and Interpersonal Skill Topics – White vs. People of Color** | **White**  ***n =* 214** | **People of Color**  ***n =* 84** |  |
| Networking in Prevention Science | 21% | 33% | -- |
| Initiating Community Collaborations | 28% | 45% | -- |
| Initiating Interdisciplinary Collaborations | 20% | 34% | -- |
| Stress Management, Work-Life Balance | 20% | 35% | -- |
| Time Management | 19% | 29% | -- |
| Working with Others with Different Backgrounds; Maintaining Diverse Teams | 15% | 28% | -- |
| Maintaining Motivation with Obstacles | 17% | 32% | -- |
| Developing and Maintaining Collaborations | 13% | 29% | -- |
| Receiving Constructive Feedback | 14% | 26% | -- |

***Note***: The n listed for each demographic group is the maximum number of respondents (of that type) who responded to any one topic in this section.

| **Communication-related Topics – Career Level** | **Early**  ***n* = 182** | **Very Likely**  **Mid**  ***n* = 66** | **Senior**  ***n* = 50** |
| --- | --- | --- | --- |
| Communicating your Work to the General Public via Various Social Media Platforms | 34% | 35% | 16% |
| Communicating with Government Officials | 42% | 27% | 14% |
| Communicating Research to Lay Audiences | 36% | 30% | 18% |
| Communicating with Foundations | 34% | 20% | 16% |
| Communicating your Work to Peers, Colleagues, or Funders via Various Social Media Platforms | 27% | 23% | 18% |
| Writing Manuscripts for Peer Reviewed Journals | 29% | 12% | 6% |
| Presenting at Professional Conferences | 21% | 8% | 10% |
|  | **Very Likely** | |  |
| **Communication-related Topics – White vs. People of Color** | **White**  ***n* = 214** | **People of Color**  ***n* = 83** |  |
| Communicating your Work to the General Public via Various Social Media Platforms | 28% | 39% | -- |
| Communicating with Government Officials | 30% | 45% | -- |
| Communicating Research to Lay Audiences | 27% | 45% | -- |
| Communicating with Foundations | 23% | 39% | -- |
| Communicating your Work to Peers, Colleagues, or Funders via Various Social Media Platforms | 20% | 35% | -- |
| Writing Manuscripts for Peer Reviewed Journals | 18% | 33% | -- |
| Presenting at Professional Conferences | 12% | 29% | -- |

***Note***: The n listed for each demographic group is the maximum number of respondents (of that type) who responded to any one topic in this section.

| **Project Management Topics – Career Level** | **Early**  ***n =* 180** | **Very Likely Mid**  ***n =* 66** | **Senior**  ***n =* 49** |
| --- | --- | --- | --- |
| Understanding Potential Funding Opportunities | 46% | 26% | 18% |
| Evaluating Project Outcomes | 38% | 31% | 12% |
| Effective Leadership | 35% | 21% | 10% |
| Developing and Managing Budgets | 38% | 20% | 4% |
| Understanding Contract/Grant Requirements | 39% | 18% | 6% |
| Developing and Managing Schedules, Timelines, Expectations, Deliverables, and Quality | 40% | 21% | 4% |
| Recruiting, Hiring, Managing, and Mentoring Project Staff | 32% | 20% | 4% |
| Meeting Management | 24% | 18% | 6% |
|  | **Very Likely** | |  |
| **Project Management Topics – White vs. People of Color** | **White**  ***n =* 213** | **People of Color**  ***n =* 82** |  |
| Understanding Potential Funding Opportunities | 35% | 42% | -- |
| Evaluating Project Outcomes | 29% | 43% | -- |
| Effective Leadership | 23% | 42% | -- |
| Developing and Managing Budgets | 23% | 44% | -- |
| Understanding Contract/Grant Requirements | 24% | 43% | -- |
| Developing and Managing Schedules, Timelines, Expectations, Deliverables, and Quality | 26% | 41% | -- |
| Recruiting, Hiring, Managing, and Mentoring Project Staff | 20% | 35% | -- |
| Meeting Management | 18% | 27% | -- |

***Note***: The n listed for each demographic group is the maximum number of respondents (of that type) who responded to any one topic in this section.

|  | **Likely Attend** | | | | **Method Unknown** | | | |
| --- | --- | --- | --- | --- | --- | --- | --- | --- |
| **Top 10 Quantitative Analysis Topics – Career Level** | **Early**  ***n* = 167** | **Mid**  ***n* = 52** | | **Senior**  ***n* = 29** | **Early**  ***n* = 167** | **Mid**  ***n* = 52** | | **Senior**  ***n* = 29** |
| Cost Effectiveness Methods | 66% | 62% | | 52% | 13% | 12% | | 0% |
| Statistical Power Analysis | 56% | 54% | | 38% | 4% | 0% | | 7% |
| Intensive Longitudinal Data Analysis | 60% | 54% | | 31% | 9% | 4% | | 3% |
| Causal Inference | 55% | 56% | | 41% | 13% | 15% | | 7% |
| Mixture Models | 55% | 54% | | 52% | 16% | 17% | | 7% |
| Propensity Score Methods | 56% | 48% | | 45% | 8% | 8% | | 3% |
| Mixture Models | 53% | 54% | | 31% | 7% | 8% | | 10% |
| Growth Modeling | 49% | 48% | | 55% | 10% | 10% | | 3% |
| Meta-Analysis | 56% | 39% | | 35% | 5% | 8% | | 3% |
| Missing Data Analysis | 56% | 39% | | 35% | 5% | 12% | | 3% |
|  |  | | | |  | | | |
|  | **Likely Attend** | | | | **Method Unknown** | | | |
| **Top 10 Quantitative Analysis Topics – Career Level – White vs. People of Color** | **White**  ***n* = 176** | | **People of Color**  ***n* = 69** | | **White**  ***n* = 176** | | **People of Color**  ***n* = 69** | |
| Cost Effectiveness Methods | 60% | | 74% | | 11% | | 15% | |
| Statistical Power Analysis | 51% | | 62% | | 3% | | 6% | |
| Intensive Longitudinal Data Analysis | 56% | | 57% | | 6% | | 12% | |
| Causal Inference | 55% | | 52% | | 11% | | 19% | |
| Propensity Score Methods | 54% | | 55% | | 14% | | 22% | |
| Mixture Models | 49% | | 64% | | 7% | | 10% | |
| Analysis of Small Sample Data | 47% | | 64% | | 9% | | 6% | |
| Growth Modeling | 47% | | 57% | | 9% | | 12% | |
| Meta-Analysis | 46% | | 57% | | 4% | | 12% | |
| Missing Data Analysis | 47% | | 58% | | 6% | | 9% | |
