# Supplementary material for: The Society for Prevention Research 20 Years Later: a Summary of Training Needs
Source: Prev Sci. 2020 Aug 3;21(7):985–1000. doi: 10.1007/s11121-020-01151-1 (PMC7462903; doi:10.1007/s11121-020-01151-1)
Supplement: Supplementary file 4 — (DOCX 15 kb) [file 11121_2020_1151_MOESM4_ESM.docx]

Supplementary Material

| **Method** | **Neither** | **Likely attend** | **Method unknown** | **Method unknown but would attend training** | **Likely attend among those who know** |
| --- | --- | --- | --- | --- | --- |
| Cost Effectiveness Methods | 28.9 | 59.7 | 8.0 | 3.4 | 67.4% |
| Statistical Power Analysis | 43.0 | 52.5 | 3.4 | 1.1 | 55.0% |
| Intensive Longitudinal Data Analysis | 39.5 | 52.1 | 6.5 | 1.9 | 56.8% |
| Causal Inference | 35.4 | 51.7 | 11.0 | 1.9 | 59.4% |
| Mixture Models | 40.3 | 51.7 | 6.8 | 1.1 | 56.2% |
| Propensity Score Methods | 33.1 | 51.0 | 13.3 | 2.7 | 60.6% |
| Analysis of Small Sample Data | 42.2 | 49.8 | 6.1 | 1.9 | 54.1% |
| Growth Modeling | 41.8 | 47.9 | 9.1 | 1.1 | 53.4% |
| Meta Analysis | 45.2 | 47.9 | 5.3 | 1.5 | 51.4% |
| Missing Data Analysis | 45.2 | 47.5 | 5.7 | 1.5 | 51.2% |
| Structural Equation Models | 45.6 | 46.0 | 7.2 | 1.1 | 50.2% |
| Latent Class and Latent Variable Modeling | 46.8 | 44.1 | 8.4 | 0.8 | 48.5% |
| Mediation Analysis | 46.8 | 44.1 | 7.6 | 1.5 | 48.5% |
| Multilevel Modeling | 51.0 | 43.3 | 4.9 | 0.8 | 46.0% |
| Individual Person-Level Meta-Analysis | 39.5 | 42.2 | 16.3 | 1.9 | 51.6% |
| Network Analysis | 42.2 | 42.2 | 13.3 | 2.3 | 50.0% |
| Data Mining | 46.4 | 41.8 | 11.0 | 0.8 | 47.4% |
| Bayesian Methods | 39.5 | 41.4 | 16.7 | 2.3 | 51.2% |
| Measurement Theory and Methods | 51.7 | 39.9 | 7.2 | 1.1 | 43.6% |
| Survival Data Analysis | 47.5 | 39.5 | 12.2 | 0.8 | 45.4% |
| Generalized Linear Modeling | 55.5 | 38.8 | 4.2 | 1.5 | 41.1% |
| Item Response Theory | 48.7 | 37.6 | 12.2 | 1.5 | 43.6% |
| Subgroup Analysis | 53.2 | 36.9 | 9.1 | 0.8 | 40.9% |
| General Linear Modeling | 60.5 | 36.5 | 2.7 | 0.4 | 37.6% |
| Moderation Analysis | 55.9 | 34.6 | 8.0 | 1.5 | 38.2% |
| Psychometric Methods | 57.0 | 34.2 | 8.0 | 0.8 | 37.5% |
| Geospatial Analysis | 39.9 | 33.8 | 23.6 | 2.7 | 45.9% |
| Survey Data Analysis | 61.2 | 32.7 | 5.7 | 0.4 | 34.8% |
| Non-Parametric Statistics | 55.5 | 31.2 | 12.2 | 1.1 | 36.0% |
| Integrative Data Analysis | 37.3 | 30.4 | 30.0 | 2.3 | 44.9% |
| Decision Analysis | 38.8 | 28.5 | 30.4 | 2.3 | 42.4% |
| Econometric Methods | 41.4 | 24.0 | 31.6 | 3.0 | 36.6% |
| Complier Average Causal Effect | 22.4 | 23.2 | 51.0 | 3.4 | 50.8% |
| Simulation Methods | 58.6 | 21.7 | 18.3 | 1.5 | 27.0% |
| System Dynamics | 43.0 | 21.7 | 33.8 | 1.5 | 33.5% |
| N of 1 Experiments | 56.3 | 17.9 | 23.6 | 2.3 | 24.1% |
| Agent Based Modeling | 20.5 | 16.3 | 60.1 | 3.0 | 44.3% |
| Analysis of High-Dimensional Data | 22.1 | 12.9 | 62.4 | 2.7 | 37.0% |
| Systems Engineering Methods | 40.7 | 12.5 | 45.2 | 1.5 | 23.6% |
| Microsimulation Methods | 40.7 | 11.0 | 46.4 | 1.9 | 21.3% |
| Genome-Wide Statistical Analysis | 49.0 | 8.7 | 39.5 | 2.7 | 15.1% |
|  |  |  |  |  |  |
| * N = 263 indicated that they would likely attend training in some area of quantitative methods | | | | |  |
| ** Values in the table indicate valid percentages (i.e., denominator is 263) | | | |  |  |
