# Supplementary material for: The Society for Prevention Research 20 Years Later: a Summary of Training Needs
Source: Prev Sci. 2020 Aug 3;21(7):985–1000. doi: 10.1007/s11121-020-01151-1 (PMC7462903; doi:10.1007/s11121-020-01151-1)
Supplement: Supplementary file 5 — (DOCX 13 kb) [file 11121_2020_1151_MOESM5_ESM.docx]

**Supplemental**

|  | Theory | Preventive Interventions | Research Methods, Design, Evaluation | Data analysis methods (Qualitative and / or Quantitative) | Mentoring | Teaching | Communication | Practical & Interpersonal Skills | Project Management |
| --- | --- | --- | --- | --- | --- | --- | --- | --- | --- |
|  | *n* = 326 | *n* = 323 | *n* = 313 | *n* = 287 | *n* = 221 | *n* = 200 | *n* = 275 | *n* = 260 | *n* = 256 |
| Self-initiated learning | 66% | 72% | 71% | 59% | 64% | 65% | 69% | 66% | 69% |
| One-on-one mentoring | 35% | 35% | 35% | 37% | 43% | 41% | 31% | 39% | 31% |
| Webinars | 64% | 65% | 70% | 60% | 69% | 75% | 78% | 69% | 73% |
| SPR Preconference Workshop | 55% | 61% | 58% | 62% | 44% | 53% | 47% | 44% | 47% |
| One day in person workshop | 37% | 41% | 49% | 53% | 31% | 40% | 34% | 36% | 37% |
| Multi-day in person workshop | 27% | 28% | 34% | 40% | 14% | 20% | 18% | 18% | 25% |
| Experiential education | 39% | 33% | 33% | 30% | 21% | 24% | 17% | 23% | 23% |

**Note:** Anything endorsed over 60% is highlighted. Blue highlights indicate the top-endorsed modality. The green indicates the second most frequently endorsed modality, and in one case the third most frequent, as in one case the third-ranked modality also received greater than 60% endorsement.
